# Reconceptualizing transcriptional slippage in plant RNA viruses

**DOI:** 10.1128/mbio.02120-24

**Published:** 2024-09-17

**Authors:** Adrian A. Valli, María Luisa Domingo-Calap, Alfonso González de Prádena, Juan Antonio García, Hongguang Cui, Cécile Desbiez, Juan José López-Moya

**Affiliations:** 1 Centro Nacional de Biotecnología (CNB-CSIC), Madrid, Spain; 2 Center for Research in Agricultural Genomics (CRAG-CSIC-IRTA-UAB-UB), Campus UAB, Bellaterra, Spain; 3 Evolving Therapeutics SL., Parc Científic de la Universitat de València, Paterna, Spain; 4 Key Laboratory of Green Prevention and Control of Tropical Plant Diseases and Pests, Ministry of Education and College of Plant Protection, Hainan University, Haikou, Hainan, China; 5 INRAE, Pathologie Végétale, Montfavet, France; University of California at Riverside, Riverside, California, USA; University of Cambridge, Cambridge, United Kingdom

**Keywords:** RNA virus, RNA polymerases, evolution, *Potyviridae*, Ebola virus, plant viruses, polymerase slippage, transcriptional slippage, overlapping ORFs, RNA replication, viral replication

## Abstract

RNA viruses have evolved sophisticated strategies to exploit the limited encoded information within their typically compact genomes. One of them, named transcriptional slippage (TS), is characterized by the appearance of indels in nascent viral RNAs, leading to changes in the open reading frame (ORF). Although members of unrelated viral families express key proteins via TS, the available information about this phenomenon is still limited. In potyvirids (members of the *Potyviridae* family), TS has been defined by the insertion of an additional A at A_n_ motifs (*n* ≥ 6) in newly synthesized transcripts at a low frequency, modulated by nucleotides flanking the A-rich motif. Here, by using diverse experimental approaches and a collection of plant/virus combinations, we discover cases not following this definition. In summary, we observe (i) a high rate of single-nucleotide deletions at slippage motifs, (ii) overlapping ORFs acceded by slippage at an U_8_ stretch, and (iii) changes in slippage rates induced by factors not related to cognate viruses. Moreover, a survey of whole-genome sequences from potyvirids shows a widespread occurrence of species-specific A_n_/U_n_ (*n* ≥ 6) motifs. Even though many of them, but not all, lead to the production of truncated proteins rather than access to overlapping ORFs, these results suggest that slippage motifs appear more frequently than expected and play relevant roles during virus evolution. Considering the potential of this phenomenon to expand the viral proteome by acceding to overlapping ORFs and/or producing truncated proteins, a re-evaluation of TS significance during infections of RNA viruses is required.

**IMPORTANCE:**

Transcriptional slippage (TS) is used by RNA viruses as another strategy to maximize the coding information in their genomes. This phenomenon is based on a peculiar feature of viral replicases: they may produce indels in a small fraction of newly synthesized viral RNAs when transcribing certain motifs and then produce alternative proteins due to a change of the reading frame or truncated products by premature termination. Here, using plant-infecting RNA viruses as models, we discover cases expanding on previously established features of plant virus TS, prompting us to reconsider and redefine this expression strategy. An interesting conclusion from our study is that TS might be more relevant during RNA virus evolution and infection processes than previously assumed.

## INTRODUCTION

RNA viruses possess small genomes, resulting in limited coding capacity. Possibly in response to this constraint, they have evolved diverse and intricate gene expression strategies. One such strategy involves the generation of *de novo* proteins via overprinting—a process wherein specific nucleotidesubstitutions, insertions, or deletions in an existing gene prompt the expression of a novel protein from an alternative open reading frame (ORF) (1). Overprinted genes can be accessed through either transcriptional or translational mechanisms. In the former, the alternative RNA is transcribed from internal promoters (2) or through transcriptional slippage (TS) (3, 4). In the latter, the protein is produced via translation from alternative start codons or by ribosomal read-throughs and frame-shifts (4, 5). Of these mechanisms, TS is the least characterized, with only few well-defined cases described across unrelated taxonomic groups. Four examples are comprised by the expression of the phosphoprotein from paramyxoviruses (6), the large glycoprotein (GP) from *Ebolavirus* (7), and both P3N-PIPO and P1N-PISPO from potyviruses (members of the *Potyvirus* genus, *Potyviridae* family) (8–10). With the exception of the better characterized slippage events leading to the expression of paramyxoviral phosphoproteins [reviewed in (11)], the precise molecular mechanism by which TS takes place is still a matter of study (12, 13). In the case of potyviruses, for instance, it is known that viral RNA-dependent RNA polymerases (RdRPs) slip over homopolymeric runs of A_n_ (*n* ≥ 6) when copying the viral genome—or U_n_ (*n* ≥ 6) if TS occurs when copying the opposite strand. As a consequence, an extra A (or U) is added in a fraction of the newly synthesized RNA molecules so that genes overprinted in the −1/+2 frame become accessible when edited transcripts are translated. Importantly, it has been observed that viral RNAs featuring a one-nucleotide (-nt) deletion at the slippage motifs (A_n_ - > A_n-1_, with *n* ≥ 6) are also generated, albeit at very low levels (12). Moreover, these one-nt-shorter RNAs might translate the +1 frame into truncated, yet functional proteins, as observed in the case of P3N-ALT from clover yellow vein virus (ClYVV, genus *Potyvirus*, family *Potyviridae*) (14).

With more than 200 assigned representatives sorted in 12 different genera, the *Potyviridae* family is the largest and most socioeconomically relevant group of plant-infecting RNA viruses. In fact, various members of this family have been included in a top 10 list for economically important plant viruses (15), as well as in a top 10 list of plant viruses in molecular plant pathology (16). Potyvirids (members of the family *Potyviridae*) have monopartite (except those in the *Bymovirus* genus, which are bipartite), singlestranded and positive sense (+ssRNA) genomes of around 10 kb (17–19). It was assumed for a long time that potyvirid genomic RNAs translated only a single, full size, ORF to produce a large polyprotein further processed by viral-encoded proteases into the mature viral factors, usually P1, HCPro, P3, 6K1, CI, 6K2, NIa (VPg-NIaPro), NIb, and CP, with some variations mostly concentrated in the N-terminal part of the polyprotein (20). However, since 2008, it has been established that an additional ORF, known as *pipo*, is overprinted in the −1/+2 frame of the P3 coding region. This ORF is preceded by a conserved GA_6_ motif and is expressed as a fusion to the N-terminal half of P3, resulting in the production of P3N-PIPO (21), a protein required for viral movement between cells (22, 23). Few years later, two independent groups discovered that the *pipo* ORF is accessed in potyviruses through TS at the GA_6_ motif (8, 9). Subsequent investigations have revealed the existence of another overprinted ORF, referred to as *pispo*, located in the −1/+2 frame of the P1 coding sequence in potyviruses infecting sweet potato (24, 25). As for *pipo*, this ORF is accessed by TS at a conserved GA_6_ motif. Consequently, the edited transcript is translated as a fusion to the N-terminal half of P1, resulting in the production of P1N-PISPO—a protein exhibiting RNA silencing suppression activity (8, 10, 26).

Among well-defined examples of TS in RNA viruses, the expression of overprinted genes in potyvirids stands out as a unique opportunity to study this phenomenon. Currently, only a limited number of TS events in viruses belonging to the *Potyvirus* genus within the *Potyviridae* family have been experimentally validated. Based on reported data, TS in plant RNA viruses exhibits the following characteristics: (i) it is observed in potyviruses and is expected to occur in all members of the *Potyviridae* family due to the conservation of the *pipo* ORF embedded in P3 coding sequences; (ii) it depends on the presence of a GA_n_ (*n* ≥ 6) motif; and (iii) it more frequently results in a single-nucleotide insertion (SNI; A_n_ -> A_n+1_, with *n* ≥ 6) than a single-nucleotide deletion (SND; A_n_ - > A_n-1_, with *n* ≥ 6). Our study, which encompasses the analysis of TS in various potyvirids from both the *Ipomovirus* and *Potyvirus* genera, and even in the unrelated potato virus X (PVX, *Potexvirus* genus, *Alphaflexiviridae* family), expands our understanding of this mechanism. Overall, our results highlight the huge flexibility of RNA viruses in exploring the expression of alternative and truncated ORFs by exploiting TS.

## RESULTS

### An atypical TS in the ipomovirus CocMoV

**Fig 1:**
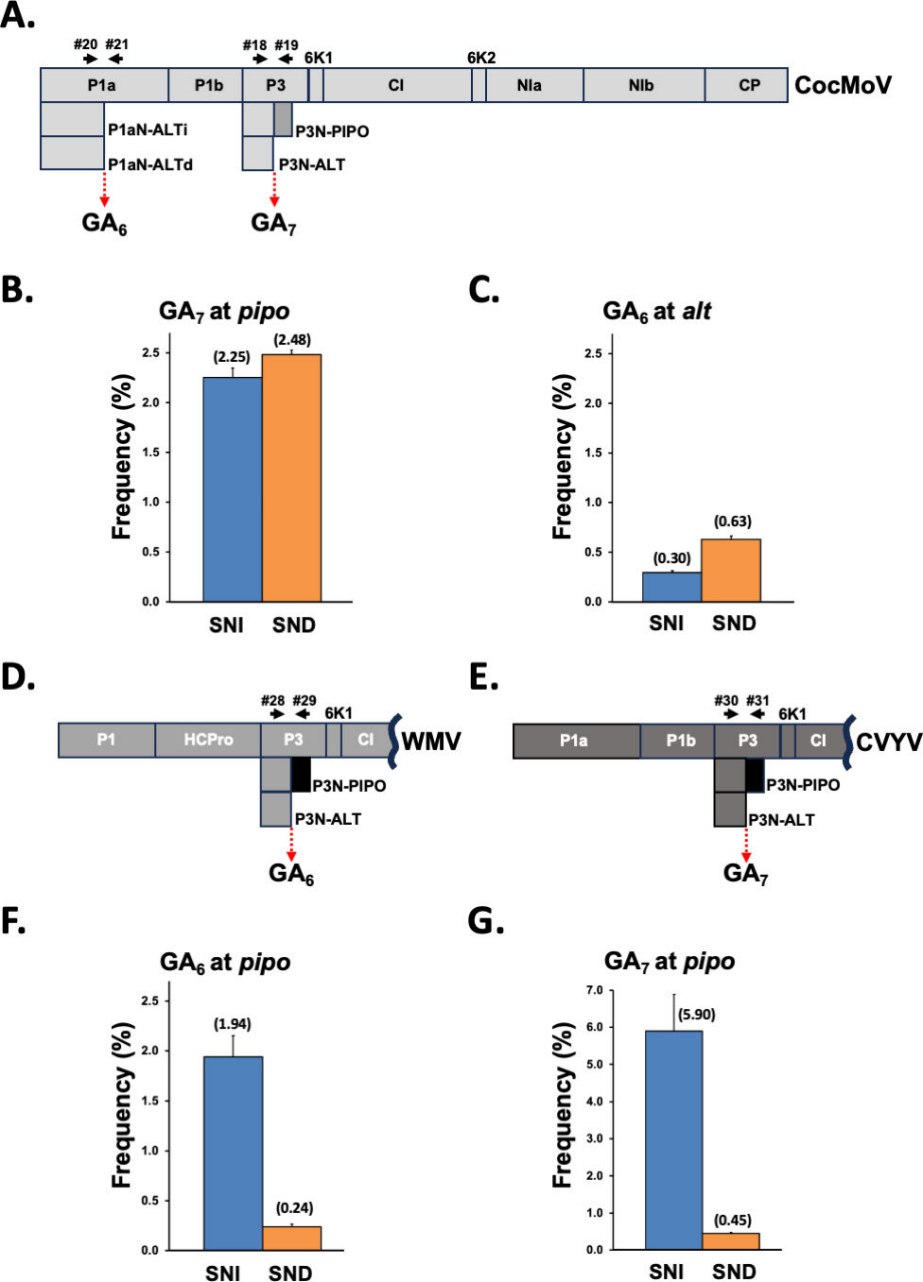
Slippage rates of potyvirids from different genera in melon plants. (**A**) Schematic representation of the CocMoV coding sequence. (**B**) Slippage rates at the GA_7_ motif located upstream of the *pipo* ORF from CocMoV. (**C**) Slippage rates at the GA_6_ motif in *alt* from CocMoV. (**D**) Schematic representation of a WMV partial coding sequence. (**E**) Slippage rates at the GA_6_ motif located upstream of the *pipo* ORF from WMV (bottom panel). (**F**) Schematic representation of a CVYV partial coding sequence (upper panel). (**G**) Slippage rates at the GA_7_ motif located upstream of the *pipo* ORF from CVYV (bottom panel). Bars represent the average frequencies (indicated between brackets) for single-nucleotide insertions (SNIs) and single-nucleotide deletions (SNDs) at the indicated slippage motifs. Error bars represent standard deviation (*n* = 3). Primers used to get amplicons are represented with black arrows in the virus map.

Although the GA_6_ slippage motif is present in the central region of the P3 coding sequence of all viruses in the *Potyviridae* family (very few slightly different sequences were recently reported, see below), empirical demonstration of TS has only been reported in members of the *Potyvirus* genus, such as plum pox virus (PPV), ClYVV, turnip mosaic virus (TuMV), sweet potato feathery mosaic virus, bean common mosaic virus, and bean common necrotic mosaic virus (8–10, 14, 26). This lack of TS evidences in viruses from other genera, as well as the presence of an additional GA_6_ motif in its P1 coding sequence (Fig. 1A), prompted us to analyze TS in CocMoV, which belongs to the *Ipomovirus* genus in the family *Potyviridae*. To do that, symptomatic upper non-inoculated leaves of melon plants infected with a natural isolate of coccinia mosaic virus (CocMoV) were harvested at 20 days post-inoculation (dpi), and their RNAs were further used to get amplicons spanning both slippage motifs by reverse transcription polymerase chain reaction (RT-PCR) (Fig. 1A). It is worth noting here that, unlike in the case of *pipo*, if insertional slippage happens at the GA_6_ motif located in the P1 coding sequence, it will not get access to an overprinted ORF due to a stop codon just downstream of the GA_6_ motif in that alternative frame (Fig. 1A; Fig. S1). Instead, a truncated version of P1a, hereafter called P1aN-ALTi (where i refers to insertion) for its similarity with the truncated P3N-ALT from ClYVV (14), would be translated.

The NGS analysis of amplicons covering the GA_6_ motif upstream of *pipo* ORF in CocMoV revealed that insertional slippage, detected by the presence of an additional A at the slippage motif (GA_6_ -> GA_7_), is observed in this ipomovirus at 2.30% of the sequenced molecules (Fig. 1B). Surprisingly, this analysis also revealed that a similar number of molecules, 2.48%, suffered deletional slippage, observed as the loss of one A at this position (GA_6_ -> GA_5_) (Fig. 1B). Although SND at the slippage motif has already been reported in potyviruses, they were clearly underrepresented in all cases when compared with SNI and even considered the result of possible artifacts produced during the preparation of amplicons (9).

The analysis of TS at the additional GA_6_ motif in CocMoV, located in the P1 coding sequence (Fig. 1A), allowed us to test whether slippage also takes place at a different position of the viral genome and, if so, whether SND and SNI are represented by similar frequencies as found at the motif that precedes the *pipo* ORF. At this particular site, we also found slippage, but at a lower overall frequency: 0.93% (Fig. 1C). Remarkably, species with SNI represented just 0.30% of the sequenced molecules, whereas those with SND represented 0.63% (Fig. 1C). Since these two species would result in two truncated versions of P1a after translation, we have named these products P1aN-ALTi and P1aN-ALTd (where d refers to deletion) (Fig. 1A; Fig. S1).

To test whether such a high frequency for SND in CocMoV was a distinctive feature of this particular virus, as well as to discard potential artifacts, we also inoculated melon plants with other two potyvirids, watermelon mosaic virus (WMV, genus *Potyvirus*) and cucumber vein yellowing virus (CVYV, genus *Ipomovirus*), in order to estimate TS. Upper noninoculated leaves of infected plants were harvested at 24 and 10 dpi for WMV and CVYV, respectively, and their RNAs were used to get amplicons spanning their corresponding slippage motifs upstream of the *pipo* ORF by RT-PCR, which were further subjected to NGS. As expected, TS events were detected in both viruses at this position. In the case of WMV, the rate of SNI was 1.94%, which was eight times higher than the 0.24% rate of SND (Fig. 1D). A similar result was observed in CVYV, which belongs to the same genus as CocMoV. In this study, the overall slippage was higher: 5.90% for SNI and 0.45% for SND, resulting in an SNI 13 times higher than that of SND (Fig. 1E). Therefore, we conclude that TS in CocMoV differs from other potyvirids in having a high frequency of SND at slippage sites, which is equivalent, or even higher, than that of SNI.

### The atypical TS in CocMoV is not the consequence of nucleotides flanking slippage motifs

**Fig 2:**
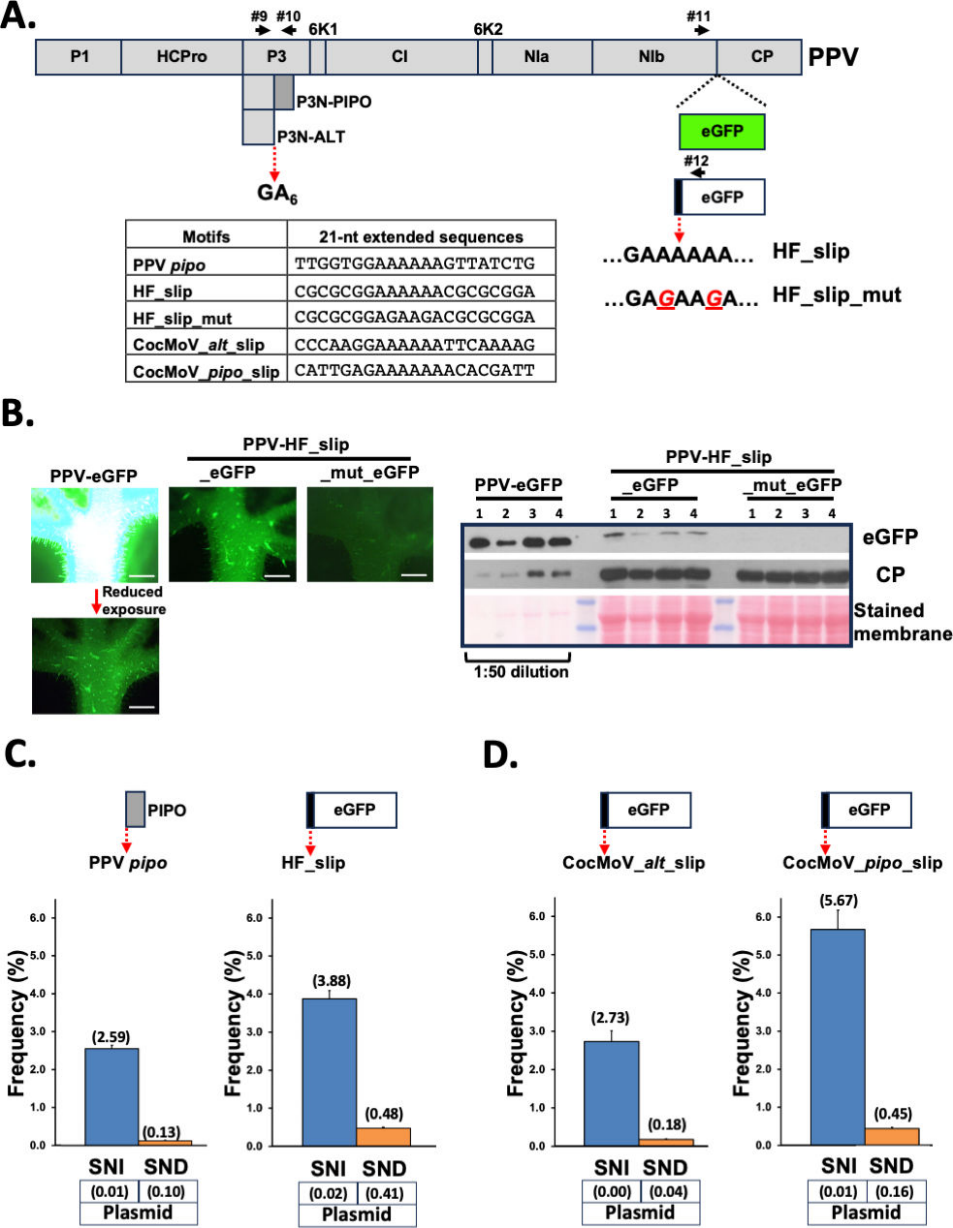
Expression of an overprinted reporter gene via TS at diverse motifs in the context of PPV infection. (**A**) Schematic representation of the PPV coding sequence. The insertion of eGFP and the out-of-frame eGFP coding sequences in the virus genome are shown. The 21-nt slippage motifs introduced upstream of the out-of-frame eGFP coding sequence are indicated in the table. Primers used to get amplicons are represented with black arrows. (**B**) Representative pictures taken under UV radiation at 9 days post-inoculation (dpi) of upper noninoculated *N. benthamiana* leaves infected with the indicated viruses are shown at the right. The leaf infected with PPV-eGFP had to be exposed 100 times less to UV radiation to display equivalent fluorescent signals than the leaf infected with PPV-HF_slip_eGFP (white bar = 1 mm). The left panel shows the detection of eGFP and PPV CP by immunoblotting analysis in protein samples from upper noninoculated leaves of four *N. benthamiana* plants infected with the indicated viruses. Blot stained with Ponceau red showing the large subunit of the ribulose-1,5-bisphosphate carboxylase-oxygenase is included as a loading control. Protein extract from leaves infected with PPV-eGFP had to be diluted 50 times to display comparable eGFP-derived chemiluminescent signals. (**C**) Slippage rates at the GA_6_ motif located upstream of the *pipo* ORF from PPV (left panel). Slippage rates at the HF_slip motif located upstream of the *egfp* ORF inserted in the PPV RNA (right panel). (**D**) Slippage rates at the CocMoV_*alt*_slip motif located upstream of the *egfp* ORF inserted in the PPV RNA (left panel). Slippage rates at the CocMoV_*pipo*_slip motif located upstream of the *egfp* ORF inserted in the PPV RNA (right panel). Bars represent the average frequencies (indicated between brackets) for single-nucleotide insertions (SNIs) and single-nucleotide deletions (SNDs) at the indicated slippage motifs. Error bars represent standard deviation (*n* = 3). The slippage rates obtained at each of those motifs by using an amplicon directly produced by the PCR from the cloned viral cDNA are indicated (plasmid).

In a previous study, Olspert and collaborators demonstrated that the overall slippage frequency in TuMV is affected by nucleotides flanking the minimal GA_6_ motif (12). If these nucleotides were responsible for the atypical TS in CocMoV, then RNA segments spanning the slippage sites of CocMoV would induce CocMoV-like slippage in a heterologous potyvirid. To test this hypothesis, we chose PPV, given that TS in this virus has already been studied and showed the typical SNI >> SND (8). We first tested whether PPV supports a genuine second slippage motif by introducing the most slippery, synthetic, G/C rich 21-nt sequence based on a GA_6_ central motif developed by Olspert and collaborators (named PISPO 5’ & 3’str in (12) and HF_slip in this study). This segment was introduced upstream of an out-of-frame eGFP coding sequence to generate an infectious clone that produces PPV-HF_slip_eGFP, which would express eGFP if one nucleotide insertion took place (Fig. 2A). In addition, we constructed an equivalent PPV derivative in which the “AAAAAA” in HF_slip was mutated to “AGAAGA” in order to generate an infectious clone that produces PPV-HF_slip_mut_eGFP, where no slippage would be expected. When inoculated in *N. benthamiana,* the control PPV-eGFP expressed high levels of eGFP in upper noninoculated leaves at 9 days post-inoculation (dpi), as observed by both fluorescent microscopy and GFP immunodetection, whereas PPV-HF_slip_eGFP expressed, although at low levels, this reporter, suggesting that TS was taking place (Fig. 2B; Fig. S2). In contrast, PPV-HF_slip_mut_eGFP did not display fluorescent nor chemiluminescent signals derived from eGFP (Fig. 2B; Fig. S2), supporting the idea that TS was abolished when the A_6_ stretch is mutated. NGS of RT-PCR products spanning the motifs upstream of the *egfp* ORF confirmed these observations: slippage was not observed at HF_mut_slip (not shown), but clearly detected at the HF_slip motif: 3.88% for SNI and 0.48% for SND (Fig. 2C). As expected from previous study results (8), the same trend with SNI >> SND was observed at the natural slippage motif located upstream of the *pipo* ORF: 2.59% and 0.13%, respectively (Fig. 2C). With these data, we conclude that PPV is able to support the expression of two independent out-of-frame products via genuine TS. NGS analyses of amplicons made by PCR directly with the plasmid as the template (negative control) showed barely detectable levels of SNI, and very low levels of SND, which were comparable to those observed in RT-PCR products, especially in the HF_slip motif (Fig. 2C). Very low but still detectable rates of SND in PCR products prepared directly from plasmids have been previously reported, suggesting that SND events occur at a low frequency during amplicon preparation and sequencing (12).

Finally, we replaced the HF-slip motif that precedes the *egfp* ORF in PPV-HF_slip_eGFP by two independent segments of 21 nucleotides corresponding to both CocMoV slippage sites. Upper noninoculated leaves of infected *N. benthamiana* plants were harvested at 9 dpi, and their RNAs were used to get amplicons spanning the motifs preceding the *egfp* ORFs by RT-PCR. Analyses of SNI and SND rates indicated that both slippage motifs from CocMoV promoted, in the context of a PPV infection, much more SNI than SND (Fig. 2D), ruling out the possibility that the atypical TS in CocMoV is due to nucleotide sequences flanking the slippage motifs. NGS of PCR products prepared from plasmids (negative controls) showed, once again, just very low levels of SND (Fig. 2D). As in the context of CocMoV infection (Fig. 1B and C), the overall slippage frequency at the motif upstream the *pipo* ORF was higher than that at *alt*. Remarkably, it was even higher than that at the HF_slip motif, which was indeed selected for this study as it promoted the highest slippage frequency out of many other tested motifs (12). These differences can be explained by the direct correlation between the slippage frequency and the length of homopolymeric runs of As (A_7_ for CocMoV_pipo_slip, and A_6_ for CocMoV_alt_slip and HF_slip), as observed for a modified slippage motif that carries either six, seven, or eight consecutive As (12).

### The atypical TS in CocMoV can be attributed to particular features of CocMoV

Based on results described above, we hypothesized that intrinsic features in CocMoV unrelated to its genomic RNA sequence define its peculiar TS. To test this hypothesis, we carried out the reciprocal experiment to the one showed in Fig. 2 by modifying the CocMoV genome in order to introduce additional slippage motifs that could induce much more SNI than SND. First, we built an infectious cDNA clone for CocMoV suitable to conveniently manipulate the viral genome (see *Materials and Methods* for more details). Zucchini and melon plants (*n* = 6 per plant species) were inoculated with the generated infectious clone by bombardment, and the infection process was monitored over the time. All plants started to display the expected symptoms of CocMoV infection in upper noninoculated leaves at 10 dpi, like the natural isolate, and the presence of CocMoV in these tissues was confirmed by RT-PCR (data not shown).

**Fig 3:**
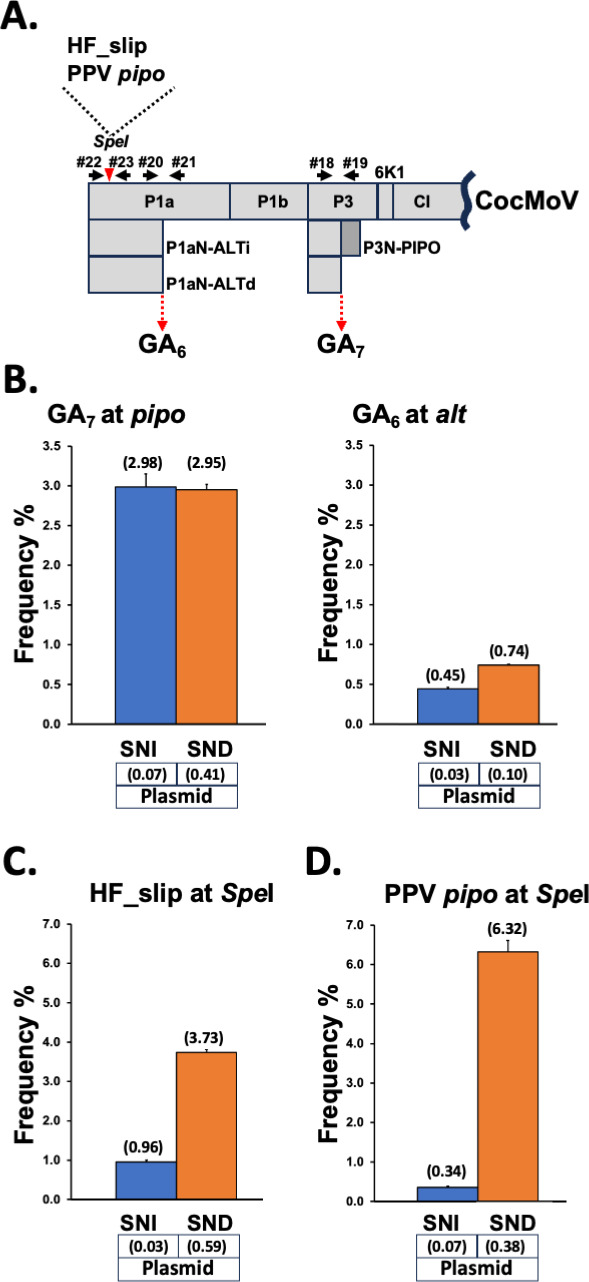
TS promoted by artificial slippage motifs in the context of CocMoV infection. (**A**) Schematic representation of a CocMoV partial coding sequence. The unique SpeI restriction site, in which the indicated 21-nt slippage motifs were introduced, is indicated with a red triangle. Primers used to get amplicons are represented with black arrows. (**B**) Slippage rates at the GA_7_ motif located upstream of the *pipo* ORF from CocMoV (left panel). Slippage rates at the GA_6_ motif located upstream of *alt* in CocMoV (right panel). (**C**) Slippage rates at the HF_slip motif inserted in the unique SpeI site of the CocMoV coding sequence (**D**) Slippage rates at the PPV *pipo* motif inserted in the unique SpeI restriction site of the CocMoV coding sequence. Bars represent the average frequencies (indicated between brackets) for single-nucleotide insertions (SNIs) and single-nucleotide deletions (SNDs) at the indicated slippage motifs. Error bars represent standard deviation (*n* = 3). The slippage rates obtained at each of those motifs by using an amplicon directly produced by PCR from the cloned viral cDNA are indicated (plasmid).

The CocMoV infectious clone was further manipulated to independently introduce, in the P1 coding sequence, and in frame with the main virus ORF, 21-nt fragments comprising the HF_slip motif and the slippage sequence upstream of the PPV *pipo* ORF (Fig. 3A). Melon plants inoculated with these modified clones displayed symptoms in upper tissues at a similar time and with the same intensity as those of plants inoculated with the wild-type CocMoV clone (data not shown). Symptomatic upper noninoculated leaves of melon plants infected with CocMoV derivatives were harvested at 20 dpi, and their RNAs were used to get amplicons spanning diverse slippage motifs by RT-PCR, which were further subjected to NGS. The analysis of natural slippage motifs in the CocMoV derivative that carries HF_slip at the 5’ end of its genome confirmed our results with the wild-type virus (Fig. 1B and C), as rates for SNI and SND were comparable at the GA_7_ motif that precedes the *pipo* ORF (Fig. 3B), whereas the SND rate was higher than that of SNI at the GA_6_ motif located at the 3’ end of *p1an-alt*. Importantly, slippage rates produced at the HF_slip introduced in the 5’ end of the CocMoV genome, contrary to what we found in the context of PPV infection, were similar to those at the CocMoV GA_6_ motif in the 3’ end of *p1an-alt*, displaying SND >>SNI (Fig. 3C). A more remarkable difference between SND and SNI was observed with a CocMoV derivative that carries, at the 5’ end of its genome, the slippage motif located upstream of the PPV *pipo* ORF (Fig. 3D). We also analyzed by NGS equivalent amplicons obtained directly by PCR reactions using plasmids as the template (negative controls). Similar to what we observed in amplicons obtained directly from PPV-based plasmids (Fig. 2C and D), small rates of SND were detected (Fig. 3C and D), but these cannot explain, in any way, the high levels of SND observed in amplicons obtained from infected plants by conducting RT-PCR. Having in mind that CVYV, a close relative of CocMoV that shares a similar host range and belongs to the same *Potyviridae* genus (*Ipomovirus*), displays the typical SNI >> SND when infecting melon (Fig. 1E), the most likely explanation for the peculiar TS found in CocMoV is that its viral polymerase is more prone to delete one nucleotide when replicating homopolymeric runs of As. As far as we know, this viral feature has not been described before.

### Wide distribution of diverse slippage motifs in the family *Potyviridae*

It has been already shown in a potyvirus, ClYVV, that a truncated P3 protein, produced from transcripts carrying 1-nt deletion in the *pipo* A_6_ stretch, is required for viral movement (14). This result, along with our unexpected findings with CocMoV, supports the idea that not only overlapping ORFs acceded by single-nucleotide insertion have to be considered potential functional products but also overlapping ORFs acceded by single-nucleotide deletion, as well as truncated versions of proteins harboring coding sequences with homopolymeric runs of As. Additionally, in view of previous results from Olspert and collaborators showing that an artificial slippage motif with U_6_ (instead of A_6_) also induces TS in TuMV (12), it seems likely that the number of functional factors produced by viruses of the *Potyviridae* family is underestimated.

Based on the above premises, we searched for A_n_/U_n_ (*n* ≥ 6) stretches in the coding sequence of fully sequenced individuals in the *Potyviridae* family (*n* = 189 species in the latest ICTV release, 13th of September 2023, https://ictv.global/vmr). Sequence features are summarized in Table S1. A first remarkable observation is that only two potyvirids, Brugmansia suaveolens mottle virus (BsMoV, GenBank accession AB551370) and Johnson grass mosaic virus (JGMV, GenBank accession Z26920), do not have any A_n_/U_n_ (*n* ≥ 6) string in their genomes. However, after careful inspection, we found that they do harbor a well-defined overlapping *pipo* ORF embedded in their P3 coding sequence (data not shown), which is preceded in both cases by GA_5_U instead of the common GA_6_ motif. The analysis of available genome sequences of more recently sequenced JGMV isolates (GenBank accessions MZ405658, KT833782, and KT289893) show, instead, that a GA_6_ motif is located upstream of their *pipo* ORF. On the other hand, and thanks to Alice Kazuko Inoue-Nagata and her team, we were able to revisit the original Sanger sequencing data of the only reported isolate of BsMoV (27). We found that two out of the three sequenced clones harboring the RT-PCR product from this virus have, indeed, GA_5_U upstream of the *pipo* ORF, but the remaining one (deposited recently with GenBank accession OR497357) has a GA_6_ slippage motif at that position. Given that the genome sequence of BsMoV, as well as that of the first reported JGMV, was assembled from few cloned RT-PCR products (27, 28), we envisage two putative scenarios to explain the lack of slippage motifs preceding the *pipo* ORF in the reported full-length sequences of these two viruses: (i) the presence of GA_5_U is simply an artifactual mutation introduced during RT-PCR, or (ii) plants might be infected with a mix of viruses in some cases, including variants without the conserved GA_6_ slippage motif upstream of the *pipo* ORF. If the second option were true, then further studies would be required to understand the biological meaning of it.

Another observation is that slippage motifs preceding the *pipo* ORF in nine out of 189 fully sequenced potyvirids are either CA_6_ (six cases) or UA_6_ (three cases) instead of GA_6_ (Table S1). Moreover, two recently reported potyvirids, tentatively named stylo mosaic-associated virus 1 (StyMaV1) and StyMaV2, not yet included in the last list released by ICTV, also have CA_6_ and UA_6_ slippage motifs, respectively, upstream of the *pipo* ORF (29). Overall, these findings indicate that G at the first position of functional slippage motifs is dispensable for TS to happen in nature.

The Table S1 also shows that, although the majority of potyvirids (130 out of 189) only have the slippage motif required to get access to the *pipo* ORF (nucleotide positions highlighted in bold and underlined), some others have additional A_n_/U_n_ (*n* ≥ 6) signatures in their genome with the potential to produce truncated forms of certain proteins in most cases (nucleotide positions are indicated) or even to get access to short alternative ORFs with more than 90 nucleotides (nucleotide positions are indicated and highlighted in bold). Additional studies will be required on a case-by-case basis to define the relevance of such additional slippage motifs over viral infections.

### A naturally occurring U_8_ motif promotes access to the *pipo* ORF

**Fig 4:**
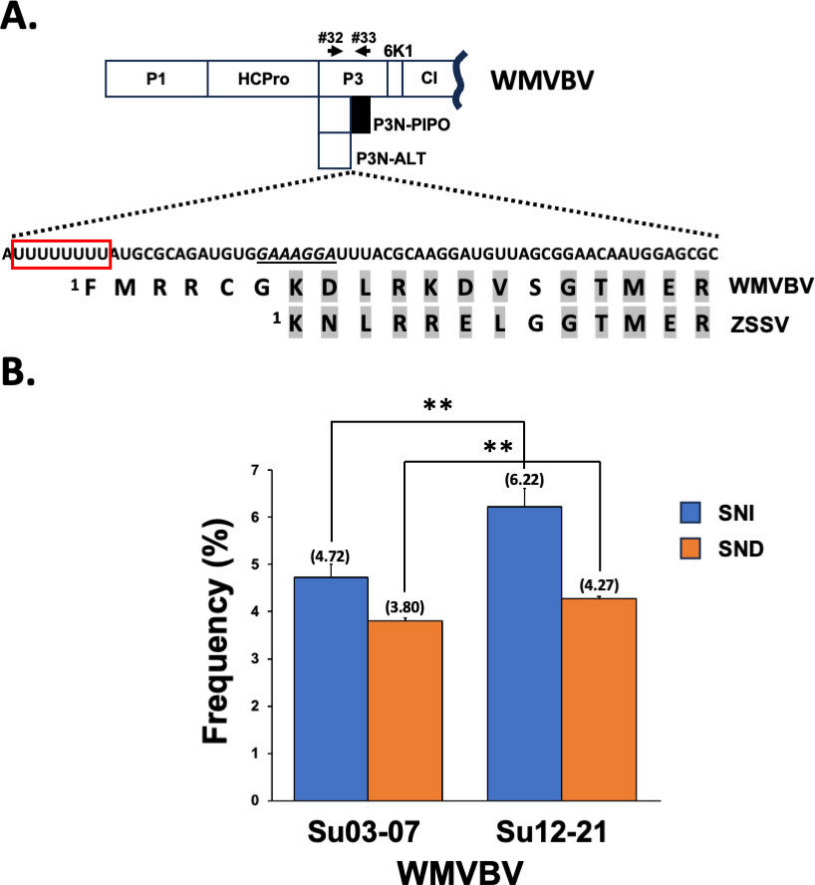
The unusual slippage motif located upstream of the *pipo* ORF in WMVBV. (**A**) Schematic representation of a WMVBV partial coding sequence. Nucleotide and amino acid sequences corresponding to the N-terminal part of PIPO in WMVBV are depicted. A sequence of nucleotides reminiscent to a GA_6_ motif is underlined. A U_8_ motif is highlighted with a red box. A partial alignment of PIPO from WMVBV and ZSV, in which equivalent/related amino acids are shadowed in gray, is shown. Primers used to get amplicons are represented with black arrows. (**B**) Slippage rates at the U_8_ motif located upstream of the *pipo* ORF from the indicated WMVBV isolates. Bars represent the average frequencies (indicated between brackets) for single-nucleotide insertions and single-nucleotide deletions. Error bars represent standard deviation (*n* = 3). Statistical differences were tested with the *post-hoc* Tukey HDS test (** *P* value < 0.01).

Surprisingly, the only available full-length genome of wild melon vein banding virus (WMVBV genus *Potyvirus*, family *Potyviridae*, isolate Su03–07) carries no A_n_ (*n* ≥ 6) motif at the P3 coding sequence. Instead, it has a U_8_ stretch located 16 nucleotides upstream of a well-defined overlapping *pipo* ORF (Fig. 4A; Table S1). We also determined the nucleotide sequence of a short fragment of the P3 coding region from Su12–21, another isolate of WMVBV for which the complete genome sequence is not available (30). A blast analysis of the WMVBV Su12–21 nucleotide sequence only retrieved a fragment of WMVBV Su03–07 and indicated that the U_8_ motif is conserved in both isolates, with an overall identity of 89% for the whole sequenced fragment (Fig. S3). Based on these findings, we hypothesized that TS might take place at the U_8_ stretch in the WMVBV genome during viral replication to get access to the *pipo* ORF. To test this idea, upper noninoculated leaves of melon plants infected with these two WMVBV isolates were harvested at 30 dpi, and amplicons spanning the slippage motif were obtained from total RNA samples by RT-PCR. NGS analysis of these amplicons revealed that, as anticipated, TS events are clearly observed for both isolates at this position (Fig. 4B). Remarkably, the rates of SNI and SND were similar in each isolate: 4.72% and 3.80% for Su03–07, and 6.22% and 4.27% for Su12–21. Differences in SNI and SND rates when comparing Su03–07 versus Su12–21 were significative (*P* value < 0.01, Fig. 4B), and it might be due to variations in the genome sequence around the slippage motif. In fact, the Su12–21 isolate, which displayed higher slippage frequencies, has two Gs just upstream of the U_8_ motif. In turn, Su03–07 has two As in these particular positions (Fig. S3), which is in perfect agreement with previous findings, indicating that increased flanking sequence GC content upstream and/or downstream of the homopolymeric run correlates with higher TS rates (12).

So far, all sequenced potyvirids, with the only exception of WMVBV, have A_6_ motifs preceding the *pipo* ORF (Table S1). In this scenario, the simplest evolutionary pathway that explains this observation is that a common ancestor of all potyvirds had A_6_ upstream of the *pipo* ORF and that the U_8_ motif evolved during WMVBV speciation. Indeed, a careful inspection of the WMVBV genome at the slippage region, and of the PIPO amino acid sequence in zucchini shoestring virus (ZSV), a virus closely related to WMVBV (18), provides some clues supporting this idea. As observed in Fig. 4A, the first amino acid in ZSV PIPO is a K, which aligned with the seventh amino acid of WMVBV PIPO. As the WMVBV coding sequence at that particular position vaguely resembles the conserved GA_6_ motif (Fig. 4A), we propose that a WMVBV ancestor evolved a U-rich stretch few nucleotides upstream of the *pipo* ORF, promoting higher slippage rates than the original GA_6_ motif, with a potential improvement in cell-to-cell movement. In fact, from all the natural TS motifs preceding the *pipo* ORF analyzed in this study, the U_8_ from WMVBV is the one displaying the highest SNI + SND rates. Another non-mutually exclusive possibility is that the presence of six extra amino acids at the N-terminus of WMVBV PIPO (FMRRCG, Fig. 4A) improves the performance of P3N-PIPO.

### Many slippage motifs in a single potyvirid

**Fig 5:**
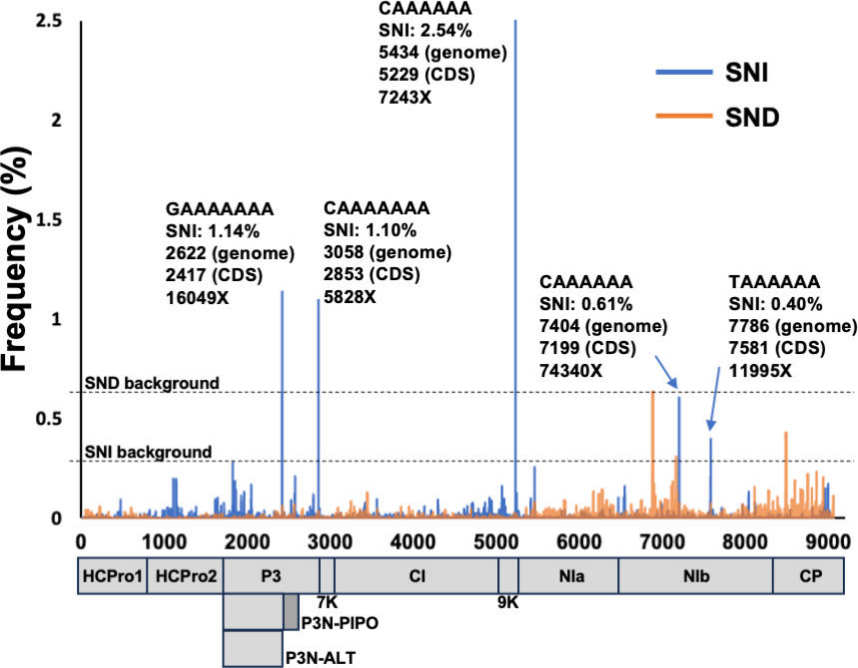
Many slippage motifs in the genome of ANSSV. Schematic representation of the ANSSV coding sequence. Rates of single-nucleotide insertions (SNIs) and single-nucleotide deletions (SNDs) in the whole ANSSV genome are shown. Slippage motifs associated to each peak along with its SNI rate, position in the viral genome and viral coding sequence, as well as the sequencing coverage at a particular position are indicated.

As shown in Table S1, areca palm necrotic spindle-spot virus (ANSSV), an exemplar from the *Arepavirus* genus, has the highest number of A_n_/U_n_ (*n* ≥ 6) motifs among potyvirids, with a total of seven. Aiming to know whether TS takes place in all of these motifs, we carried out a transcriptomic analysis of leaves from an areca palm infected with ANSSV. With a mean coverage of 34,000X per nucleotide position, we were able to estimate both SNI and SND frequencies with high confidence. For this analysis, we considered that background slippage rates for SNI and SND are 0.28% (at position 2,028) and 0.63% (at position 7,088), respectively, due to the slippage events observed in sequences different from A_n_/U_n_ (*n* ≥ 6) (positions 2,028 and 7,088) (Fig. 5). Therefore, whereas no peak above the background was observed for SND, only three positions presented a rate of SNI way above the background (Fig. 5). A careful inspection of those positions indicated that they correspond to three A_n_ (*n* ≥ 6) motifs including, as expected, the one immediately upstream of the *pipo* ORF (position 2,622, Fig. 5).

#### Even more interesting are the following findings

1. the A_7_ motif upstream of the *pipo* ORF (position 2,622) is not the one displaying the highest TS frequency. Indeed, an A_6_ motif at position 5,434 promoted higher levels of SNI (2.54% vs 1.14%, Fig. 3). This particular motif, along with another relevant A_7_ run at position 3,058 (SNI = 1.10%, Fig. 5), gives no access to overlapping ORFs so that they might be playing another role. It is relevant to mention that both motifs are located around the boundaries of two cistrons (P3-7K for the motif at position 3,058 and 9K-NIa for that at position 5,434), and one can speculate that they play a role in the regulation of polyprotein truncation at certain places via the introduction of premature stop codons;
2. the only presence of an A_n_/U_n_ (*n* ≥ 6) motif in a potyvirid is not enough for significant TS to happen. ANSSV has seven of these motifs in its genome, those mentioned in point (i), three additional A_6_ runs, and one U_6_ motif. From the last four, the A_6_ motifs at positions 7,404 and 7,786 displayed a TS frequency just above the background (SNI of 0.61% and 0.40%, respectively, Fig. 5). However, runs of A_6_ at position 5,665 and U_6_ at position 5,619 displayed a marginal SNI of 0.25% and 0.10%, respectively.

Diversity in promoting TS at different rates when comparing the seven motifs might be due to differences in the flanking nucleotide sequences, as previously shown with artificially introduced slippage motifs in TuMV (12). All in all, data presented so far highlight the sequence flexibility of natural slippage motifs (long homopolymeric runs of As or Us) and reinforce the idea that TS might take place at different positions, all along potyvirid genomes, independently of the presence/absence of downstream overlapping ORFs.

### Factors unrelated to the cognate virus shape TS rate

#### Effect of mixed infections on the TS rate

To test whether the presence of another virus alters SNI and/or SND rates in CocMoV, we performed mixed infections of CocMoV +WMV and CocMoV +cucurbit yellow stunting disorder virus (CYSDV isolate AILM, *Crinivirus* genus, *Closteroviridae* family). We harvested systemically infected leaves at 20 dpi and prepared RNA samples in order to get amplicons spanning CocMoV slippage motifs by RT-PCR, which were then subjected to NGS. We found that slippage rates at the GA_6_ motif that precedes *p1an-alt* in CocMoV were not affected by the presence of WMV (Fig. S4). On the contrary, slippage at the GA_7_ motif that precedes the *pipo* ORF was altered in the mixed infection as the presence of WMV caused a significative reduction in the SNI rate (1.86% vs 2.25%) (Fig. S4). Different results were observed in plants infected with CocMoV +CYSDV, as in this particular case, both SNI and SND were slightly higher at the GA_7_ motif that precedes the *pipo* ORF (2.86% and 2.63% vs 2.25% and 2.48%, respectively) (Fig. S4). Comparable to the case of CocMoV +WMV, TS rates at the GA_6_ motif that precedes the *p1an-alt* ORF were not significantly affected in CocMoV +CYSDV-infected plants (Fig. S4). All in all, these results are in line with our previous observation in a different pathosystem (26), thus confirming that coinfection with other viruses can affect TS rates. Even though additional experiments are required to understand the meaning of these observations, as well as mechanisms underpinning these effects, we speculate that complementation and competition occur.

#### Host effect on TS

To test whether SNI and SND rates for a given virus are somehow influenced by the host identity, we used sweet potato virus 2 (SPV2, *Potyvirus* genus, *Potyviridae* family) as model given that

**Fig 6:**
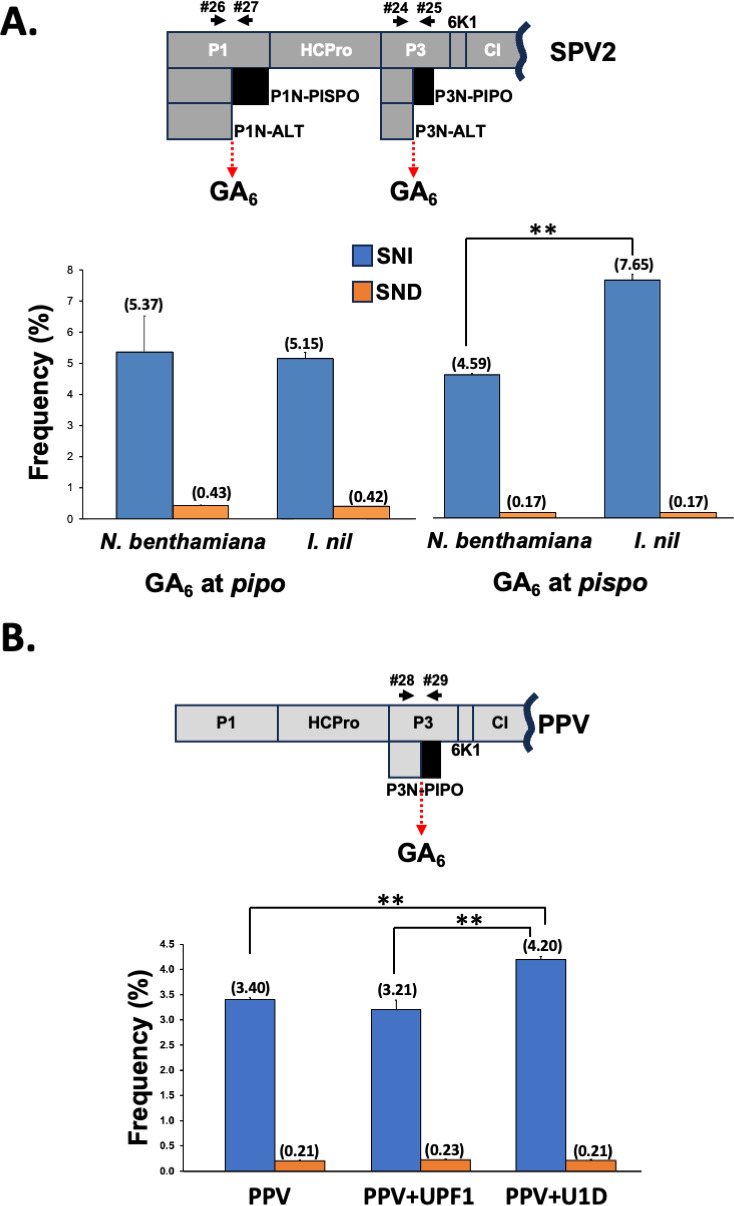
Host influences on TS rates. (**A**) Schematic representation of a SPV2 partial coding sequence, including slippage motifs and primers used to get amplicons (black arrows). Slippage rates at the GA_6_ motifs upstream both *pipo* and *pispo* ORFs from SPV2 are shown. (**B**) Schematic representation of a PPV partial coding sequence, including the slippage motif and primers used to get amplicons (black arrows). Slippage rates at the GA_6_ motif upstream of the *pipo* ORF from PPV in PPV-infected plants, supplemented in *trans* with the indicated proteins by agroinfiltration, are shown. Bars represent the average frequencies (indicated between brackets) for single-nucleotide insertions (SNIs) and single-nucleotide deletions (SNDs) at the slippage motifs. Error bars represent standard deviation (*n* = 3). Statistical differences were tested with the *post-hoc* Tukey HDS test (** *P* value < 0.01).

1. SPV2 belongs to the subgroup of sweet potato-infecting potyviruses, which encode two ORFs overlapping the main viral ORF: *pispo* in the P1 coding sequence and *pipo* in that of P3 (Fig. 6A), both acceded by TS, as demonstrated in the closely related sweet potato feathery mottle virus (SPFMV) (8, 10),
2. SPV2 is able to infected diverse hosts besides ipomeas (31), such as the model plant *N. benthamiana*.

Symptomatic upper noninoculated leaves of *Ipomea nil* (natural host) and *N. benthamiana* (laboratory host) plants infected with SPV2 were harvested at 14 dpi, and the corresponding RNA samples were used to get amplicons spanning both slippage motifs by RT-PCR (Fig. 6A). NGS analysis of amplicons showed that, as expected, TS took place during viral replication at both motifs in both hosts. Like for most viruses studied here and in previous works, SNI rates were much higher than those of SND (Fig. 6B and C). Interestingly, although TS rates at the motif located upstream of the *pipo* ORF were similar in both hosts (Fig. 6B), those at the motif that precedes the *pispo* ORF were significantly different, particularly in the case of SNI (*P* value < 0.01). SNI was more frequent in *I. nil* than in *N. benthamiana* (7.65% versus 4.59%, Fig. 6C), supporting the idea that P1N-PISPO, the protein expressed from RNA molecules carrying an additional A, accumulates at higher levels in the natural host *I. nil*. Assuming that SPV2 P1N-PISPO interferes with host RNA silencing, like its counterpart from SPFMV (10, 26), and given that the laboratory strain of *N. benthamiana* is a natural knock-out mutant of RDR1 and has lost one copy each of DCL2/3, AGO2, and RDR6, all key proteins for antiviral host-based defense mediated by RNA silencing (32–34), one can speculate that the pressure to produce P1N-PISPO is lower in this host; in this scenario, slippage at the motif that precedes the *pispo* ORF would not be as necessary in *N. benthamiana* as in other silencing-proficient plants.

#### Host-dependent non-sense-mediated RNA decay affects the TS rate

A direct consequence of TS in potyvirids is the production of viral RNAs with an exceptionally large 3’ untranslatable region (UTR). For instance, RNA species coding for P1-HCPro-P3NPIPO harbor 3’ UTRs of around 7,000 nucleotides. In all eukaryotes, coding RNAs with large 3’ UTR are commonly targeted by the non-sense-mediated mRNA decay (NMD), which is a surveillance pathway that safeguards the quality of the transcriptome by eliminating mRNAs with premature stop codons (35). In order to test whether TS products are recognized and eliminated by the NMD pathway, we infected *N. benthamiana* plants with PPV and further blocked NMD in infected, upper noninoculated leaves. Such a blockage was achieved by inhibiting UPF1, a key factor of the NMD mechanism, via the expression of the dominant negative mutant U1D (36). As controls, we used untreated infected plants, as well as plants transiently expressing the wild-type UPF1. Three days after the treatment, total RNAs were prepared from systemically infected leaves, which were further used as the template to get amplicons spanning the PPV slippage motif that precedes the *pipo* ORF by RT-PCR (Fig. 2A). NGS analysis of these amplicons showed a significant increase in the SNI rate when the NMD was inactivated (Fig. 6D). On the other hand, the analysis also indicated that the very low SND rate does not increase when expressing U1D (Fig. 6D). This result might be justified by the previously proposed idea that such low SND rates are mostly artifacts during library preparation and NGS (12).

### Transcriptional slippage outside the *Potyviridae* family

**Fig 7:**
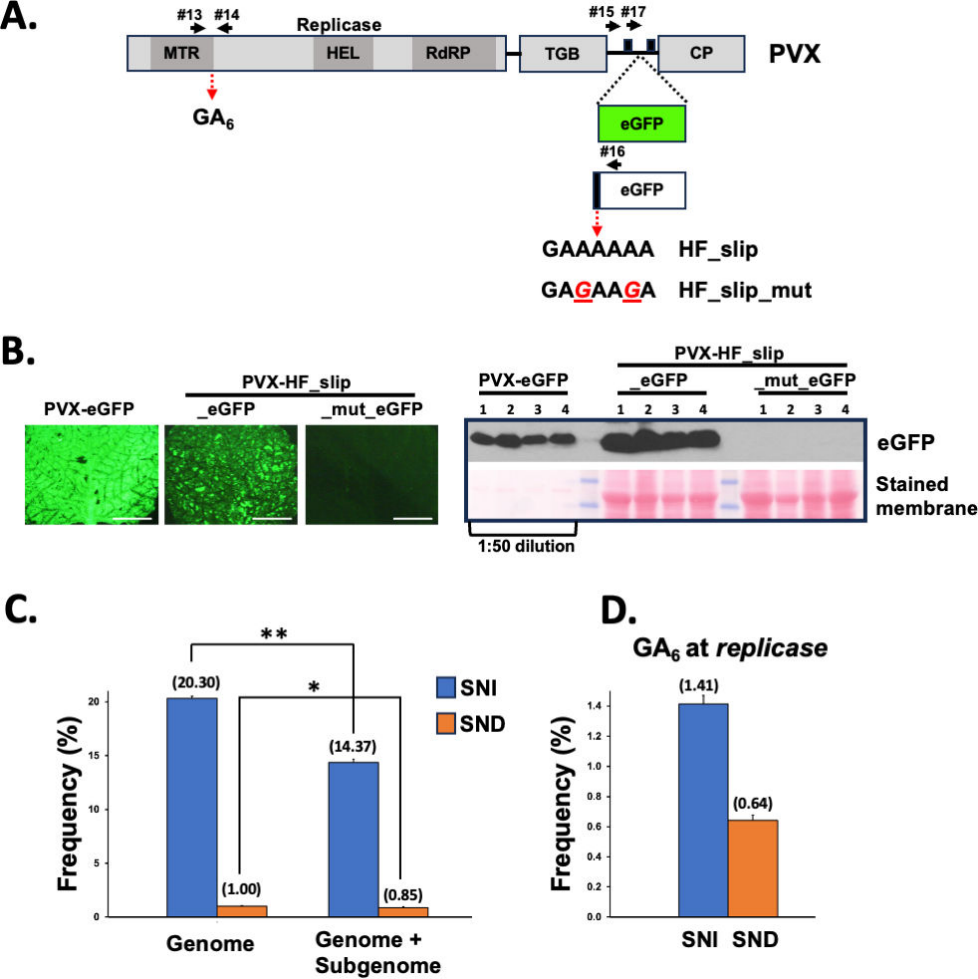
TS outside of the *Potyviridae* family, the case of PVX. (**A**) Schematic representation of the PVX coding sequence. The insertion of eGFP and the out-of-frame eGFP coding sequences in the virus genome is shown. The slippage motifs introduced upstream of the out-of-frame eGFP coding sequence are also indicated. The two copies of the subgenomic promoter are depicted as black boxes. Functional domains presented in the replicase protein are indicated (MTR: methyltransferase; HEL: helicase; RdRP: RNA-dependent RNA polymerase). Primers used to get amplicons are represented with black arrows. (**B**) Representative pictures taken under UV radiation at 9 days post-inoculation of upper noninoculated *N. benthamiana* leaves infected with the indicated viruses are shown at the right (white bar = 1 cm). The left panel shows the detection of eGFP by immunoblot analysis in protein samples from upper noninoculated leaves of four *N. benthamiana* plants infected with the indicated viruses. Blot stained with Ponceau red showing the large subunit of the ribulose-1,5-bisphosphate carboxylase-oxygenase is included as a loading control. The protein extract from leaves infected with PVX-eGFP had to be diluted 50 times to display comparable eGFP-derived chemiluminescent signals. (**C**) Slippage rates at the HF_slip motif located upstream of the *egfp* ORF inserted in the PVX RNA. (**D**) Slippage rates at the GA_6_ motif located downstream of the MTR motif from the PVX replicase coding sequence. Bars represent the average frequencies (indicated between brackets) for single-nucleotide insertions (SNIs) and single-nucleotide deletions (SNDs) at the indicated slippage motifs. Error bars represent standard deviation (*n* = 3). Statistical differences were tested with the *post-hoc* Tukey HDS test (* *P* value < 0.05; ** *P* value < 0.01).

Finally, in order to check whether TS takes place in RNA viruses unrelated to the *Potyviridae* family, we used the well-characterized potato virus X (PVX, *Potexvirus* genus, *Alphaflexiviridae* family) as the model. We manipulated a previously described PVX vector (37) to generate an infectious clone that produces PVX-HF_slip-eGFP, a virus that expresses, from a duplicated subgenomic promoter, an out-of-frame eGFP coding sequence. In this case, the reporter is preceded by an AUG (to initiate translation) followed by the HF_slip motif, the eGFP coding sequence, and a stop codon (Fig. 7A). Hence, as for PPV-HF_slip-eGFP, the eGFP would be produced during infection only if +1/–2 A occurred. In addition, we constructed an equivalent PVX derivative in which the A_6_ motif in HF_slip was replaced by AGAAGA to generate an infectious clone that produces PVX-HF_slip_mut_eGFP. These two PVX derivatives, along with PVX-eGFP, in which the eGFP coding sequence is in frame with the initiation codon (Fig. 7A), were inoculated in *N. benthamiana* plants, and systemically infected leaves were harvested at 6 dpi. As expected, PVX-eGFP expressed high levels of eGFP in upper noninoculated leaves, as observed by both fluorescent microscopy and GFP immunodetection, whereas PVX-HF_slip_eGFP expressed lower levels of this reporter, which was clearly detected by both methods (Fig. 7B; Fig. S5). In contrast, PVX-HF_slip_mut_eGFP did not display fluorescent nor inmunodetection signals derived from eGFP (Fig. 7B; Fig. S5).

The NGS analysis of RT-PCR amplicons spanning the introduced slippage motifs (primer pair #15/#16, Fig. 7A) showed background levels of SND and SNI in HF_slip_mut RNAs, confirming that TS was not taking place in this virus (data not shown). In turn, slippage rates at the HF_slip motif in PVX was even higher than that in PPV: 20.32% for SNI and 1% for SND in the PVX background, vs 3.88% for SNI and 0.48% for SND in the PPV background (Fig. 7C and 2C). Such a high TS frequency in PVX might be due to the particular strategy used to express the overlapping ORF. In the case of PVX, genome variants with +/-A at the slippage motif would retain the propagation potential and are maintained in the viral population as the ORF for the CP cistron, which is located downstream of HF_slip_eGFP, is not disrupted by the upstream editing since the CP transcript is expressed from its own subgenomic promoter (Fig. 7A). In contrast, PPV variants with +/-A at the slippage motif would tend to be eliminated from the viral population as the ORF for the CP cistron, which is also located downstream of HF_slip_eGFP, is no longer acceded when TS takes place (Fig. 2A).

To gain insights into the production of overlapping ORFs in PVX, we also carried out NGS analysis of RT-PCR amplicons spanning the introduced slippage motif, but produced with another forward primer (same RNA samples as above, primer pair #17/#16, Fig. 7A). Since this primer anneals downstream of the duplicated subgenomic promoter, the template for this amplification includes the PVX genome (as above), as well as the subgemonic RNA. As expected, TS was not detected at significant levels in the HF_slip_mut motif (data not shown), whereas the TS rate at HF_slip_eGFP was 14.37% for SNI and 0.87% for SND (Fig. 7C). Interestingly, these are not as high as slippage rates when using only the genome as the template (Fig. 7B), indicating that the accumulation of subgenomic RNA species with +/-A at the slippage motif is somehow constrained. As no difference in the transcription rate of diverse subgenomic RNA variants is anticipated, this result fits with two putative scenarios: (i) PVX polymerase is more prone to slip when replicating the viral genome than during transcription of the subgenomic RNA, or (ii) the stability of subgenomic RNA variants with +/-A at the slippage motif is lower than that of the variant without insertion/deletion. The second scenario is not the most likely as, indeed, the RNA variant with no insertion/deletion has a stop codon in the main frame just downstream the slippage motif; therefore, it is not translated (not protected/stabilized by ribosomes) and has a longer 3’ UTR (more prone to be recognized and degraded by the NMD mechanism).

Although not conserved in all reported strains, most PVX genomes annotated at the NCBI, including the one used in this study, harbor an A_6_ run at the viral replicase coding sequence, specifically at the 3’ end of the segment that codes for the methyltransferase domain (Fig. 7A). We used the above RNA samples as the template to generate RT-PCR amplicons spanning this motif (Fig. 7A) and, by analyzing the NGS data, found that TS events take place at this position, although at a lower level than at the HF_slip site (Fig. 7C). In this particular case, the so-produced viral RNA species with +/-A at the slippage motif do not give access to an overlapping ORF; instead, they produce truncated versions of the viral replicase, including only its methyltransferase domain. In fact, truncated fragments of the PVX polymerase have been detected during infection (38). Therefore, TS may play a role here in the production of a free methyltransferase, thus mimicking what other viruses do via different strategies [e.g., nsP1 from alphaviruses (39)].

## DISCUSSION

Even though the conservation of genome sequences just upstream of the PIPO coding sequence had already anticipated that *pipo* ORF was acceded via TS in all viruses from the family *Potyviridae*, empirical demonstration was just available for a few members of the *Potyvirus* genus. Our work, along with other recently published studies, indicates that TS indeed takes place not only in potyvirids from other genera but also in an unrelated plant RNA virus such as PVX, which belongs to the *Alphaflexiviridae* family [(40, 41) and this work]. Similar observations in animal-infecting RNA viruses, in both naturally occurring and artificially introduced slippage motifs, have been reported (6, 7, 13) (42). Together, these results support the idea that viral polymerase slippage is a widespread feature of RNA viruses in general. For other viruses, such as those with DNA genomes, this strategy would not be effective, as most of them are replicated/transcribed by polymerases carrying proofreading activity capable of repairing an insertion or deletion (43).

Notably, the data depicted in Fig. 1 and 3 unveil a previously underestimated phenomenon of TS in RNA viruses: the production of viral RNA species harboring a high proportion of SND at slippage motifs, comparable in frequency to those with SNI. Something similar had been reported for a paramyxovirus synthetic minigenome with mutations introduced at the slippage sequence (44). Although SND events do not provide access to overlapping ORFs in CocMoV, the elevated frequency of these particular molecules compels us to hypothesize potential roles and evolutionary implications for the production of RNA species coding for truncated proteins. Regarding roles, two non-mutually exclusive scenarios are plausible: (i) they work as a negative self-regulatory strategy to avoid viral overaccumulation and the deleterious consequences of virus diseases for the host, as truncated proteins might not be functional; (ii) they are translated into truncated, yet functional, proteins, thus enlarging the coding capacity of viral genomes. Indeed, an example that supports the feasibility for this second scenario has been reported for ClYVV (14), and it might also be the case reported here of the sole MTS domain expressed during PVX infection (Fig. 7D). An alternative option within this second scenario is exemplified by the case of ANSSV, where polymerase slippage results in RNA species with premature stop codons in close proximity to sequences encoding protease cleavage sites, thus having the potential to complement the action of viral proteases (Fig. 5).

**Fig 8:**
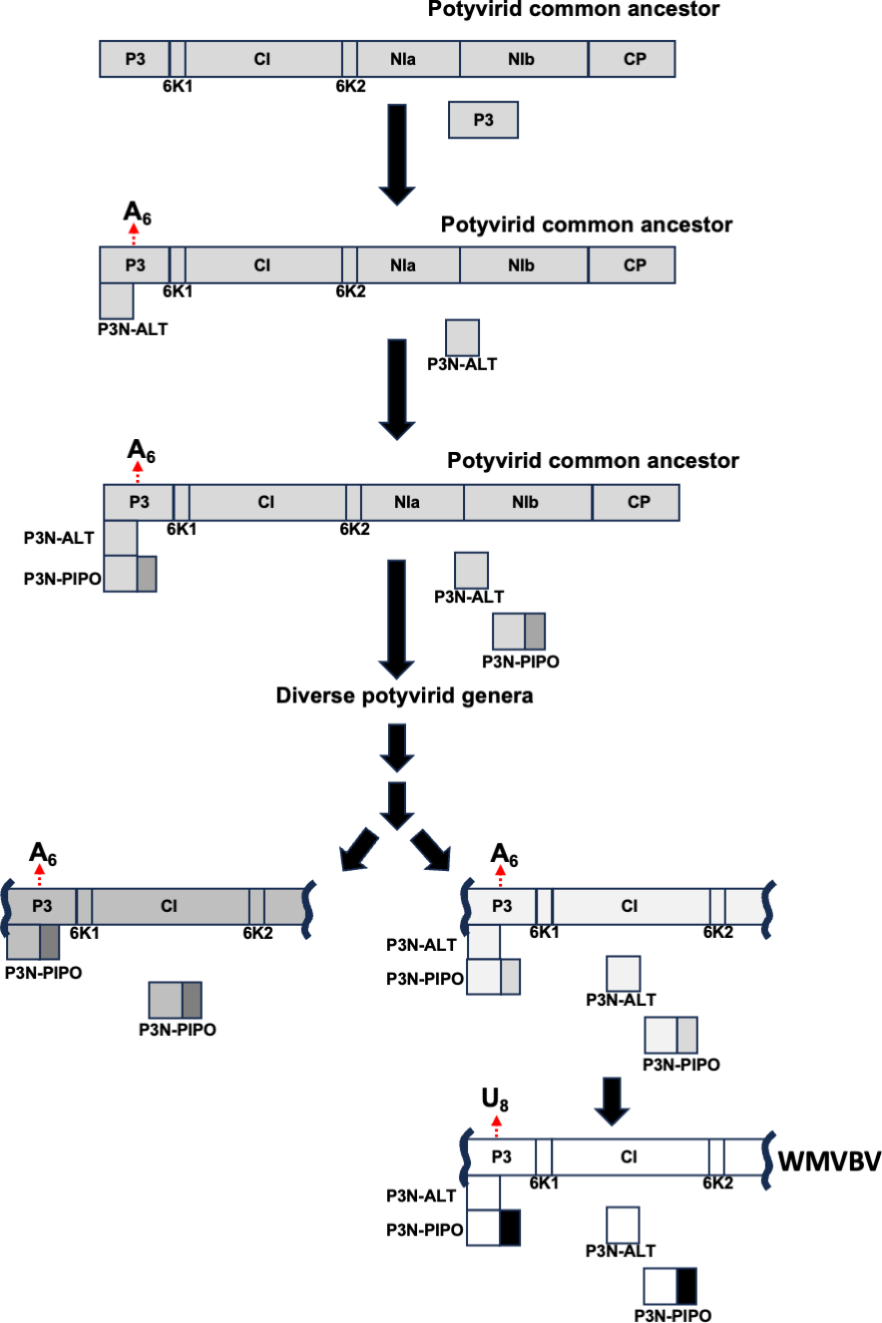
Hypothetical evolutionary trajectory of potyvirids, which explains the appearance of a slippage motif and the *pipo* ORFs in the P3 coding sequence. Schematic representation of the coding sequences from ancestral potyvirids, as well as P3-related factors associated to potyvirid cell-to-cell movement, at each step in the evolution of the *Potyviridae* family. See the main text for more information.

In terms of evolution, the production of RNAs coding for truncated proteins fits well with a parsimonious evolutionary trajectory that potentially explains the appearance of *pipo* ORF in all potyvirids (Fig. 8). It seems logical to assume that the genome of a common ancestor for all potyvirids encoded from P3 to CP, with P3 and CI playing roles in cell-to-cell movement (45). The next step would have included the appearance of a repetition of As in the central region of P3, which promoted TS (SNI/SND) and, consequently, the appearance of few viral RNA species with a premature stop codon. These peculiar RNAs, when translated, would have produced a truncated form of P3, which took over the role of P3 during virus cell-to-cell movement, thus resembling P3N-ALT from ClYVV (14). In that scenario, the hypothesized role of P3 in virus movement would have not been necessary, meaning that the 3’ half of the P3 coding sequence could evolve to either improve another former P3 role or acquire a new one. This idea, in fact, is in line with recent analyses showing that the region of potyviral genomes coding for the C-terminal part of P3, but not that coding for the N-terminal part of it, displays high variability (46). Later during evolution, from TS events induced by the homopolymeric run of As, the SNI would have been able to explore an alternative frame, then extending the size of *alt* to finally become *pipo*, which improved the role of the translated protein in virus cell-to-cell movement. After different genera evolved, some viruses seem to have specialized to mostly introduce an A at the slippage motif to get access to *pipo*, whereas others seem to still get access to *alt* and *pipo* at different ratios, depending on their ability to delete/add a single A at the slippage motif during replication. Further evolution events had to be necessary to explain the appearance of a U_8_ slippage motif (and the disappearance of A_6_) upstream of the *pipo* ORF in WMVBV (Fig. 4).

A striking conclusion from our analysis of all available potyvirid full-length genome sequences (Table S1) is that, apart from the conserved slippage motifs located upstream of *pipo* and *pispo* ORFs in all potyvirids and in a subset of potyviruses infecting sweet potato, respectively, many species-specific A_n_/U_n_ (*n* ≥ 6) motifs are present in certain viral genomes. Importantly, some of them would give access to short ORFs with more than 30 amino acids (e.g., catharanthus mosaic virus and cucurbit vein banding virus), while the others would promote the expression of truncated versions of viral proteins. Having in mind that homopolymeric runs of nucleotides formed by A_6_, A_7_, U_6,_ or U_7_ are underrepresented in potyvirid genomes (8, 9), it is then reasonable to hypothesize that these motifs were retained during evolution since TS at those positions play a role during the course of viral infections. Further analyses to detect significant evidence of purifying selection for the additional slippage motifs in these particular viruses would be required to reach a definitive conclusion on this matter. On the other hand, the analysis shown in Fig. 4 with ANSSV suggests that not all A_n_/U_n_ (*n* ≥ 6) motifs, when comparing to other sequences, seem to induce the production of RNA molecules having SNI/SND. A previous study with Ebola virus supports this idea, as it showed that the viral replicase slips over an A_7_ motif to introduce an additional A in the GP coding sequence, but not in an equivalent motif in the sequence coding for the L protein. In that report, authors demonstrated that a short loop located just upstream of the slippage motif is critical for this SNI to happen (13). In the case of potyvirids, a report based on TuMV indicated that nucleotide sequences flanking the homopolymeric run of As are also relevant; however, in that particular case, it is the GC content rather than the RNA secondary structure that influences TS (12). Moreover, as an additional layer of complexity, we also found that factors unrelated to cognate viruses, such as the coinfection with other viruses and the identity of infected hosts, also influence TS rates (Fig. 5; Fig. S4). The fact that different hosts do not accumulate the same proportion of slippage products might be due to their intrinsic capacity to prevent the accumulation of RNAs harboring premature stop codons with large 3’UTRs, such as those produced by TS. Indeed, we found strong evidences that NMD is, at least in part, preventing the accumulation of natural slippage products in *N. benthamiana* plants infected with PPV (Fig. 5B). Importantly, when we were undertaking this study, results from an independent group showed a comparable impact of NMD over the rate of slippage products in TuMV by using a different approach (47). Intriguingly, the impact of NMD on potyvirid infections remains a subject of discussion due to conflicting results (47–49). The mentioned study on TS in TuMV also indicates that SNI is slightly higher at later infection stages, and with increasing temperatures; unfortunately, the particular effect of these variables on SND in TuMV was not informed (47), likely because overall SND frequencies are very low, as previously reported for this virus (9, 12). Differences in the slippage frequency at different time points after virus inoculation were also reported for paramyxoviruses in cell cultures (50, 51). Here, we did not consider harvesting time and environmental conditions as variable. In fact, most of our results derive from treatment-vs-control comparisons in which treated and control plants were grown under identical environmental conditions in each experiment, and tissues were harvested simultaneously. The only exception is the experiments shown in Fig. 1, in which upper noninoculated leaves of melon plants independently infected with CocMoV, WMV, or CVYV were grown in identical conditions but harvested at different dpi based on viral fitness. Based on the minimal effect of infection stages on TS in TuMV (47), it is very unlikely that our interpretation about peculiar features in CocMoV slippage is incorrect due to samples being harvested at different time points.

As demonstrated in this study and others, numerous factors have the potential to influence TS rates either positively or negatively so that it appears necessary to study this phenomenon on a case-by-case basis. Nevertheless, given the impact that genuine TS has over the viral proteome, it will be exciting in the future to identify new examples of protein expression via this strategy in RNA viruses and investigate not only the reasons and consequences of TS alterations induced by diverse factors but also the mechanisms promoting these changes.

## MATERIALS AND METHODS

### Plants, virus isolates, and bacteria strains

Melon plants (cv. Védrantais) were grown in a greenhouse with 16-h/8-h light/dark cycles at 22°C to 25°C. *Nicotiana benthamiana*, *Ipomoea nil,* and *Cucurbita pepo* (cv. Diamant F1) plants were grown in a greenhouse at 24°C to 28°C (day) and 22°C to 24°C (night) with a natural photoperiod (no less than 12 h of light, supplemented with artificial illumination if needed). The *Areca catechu L*. tree was grown in the field.

Natural isolates of CocMoV (isolate Su12–25, GenBank accession no. KU935732) (52), WMVB (isolate Su03–07, GenBank accession no. KY623506) (30), and CYSDV (isolate AILM, GenBank accession nos AY242077 and AY242078) (53) were maintained in melon plants. A natural isolate of CVYV (isolate AILM, GenBank accession no. MK777994) (54) was maintained in *Cucurbita pepo*. A natural isolate of SPV2 (isolate AM-MB2, GenBank accession no. KU511270) (26) was maintained in sweet potato plants. A natural isolate of ANSSV (isolate HNBT, GenBank accession no. MH330686) (55) was identified and maintained in an *A. catechu L*. tree in the field (Hainan, China).

A previously built infectious clone of WMV (56) was used to inoculate melon plants in order to get fresh infected tissue for further manual inoculations.

*Agrobacterium tumefaciens* strains carrying either pBIN-UPF1, pBIN-U1D, or pBIN-P14 (36) were kindly provided by Prof. Daniel Silhavy (Institute of Plant Biology, Eötvös Loránd Research Network, Szeged, Hungary).

### Primers

A list of all oligonucleotides/primers used for this study, with their corresponding sequences, is shown in Table S2.

### Plasmids

PPV-derived constructs with an out-of-frame eGFP preceded by the indicated slippage motifs were built by LR recombination by using the previously described pGWBin-PPV (57) as the destination vector and the corresponding pDONR-slippage_motif_eGFP variants as entry clones. These were prepared by BP recombination between pDONR207 (Invitrogen) and synthetic DNA strings harboring (from 5’ to 3’): attB1, the corresponding 21-nt slippage motifs, the eGFP coding sequence with synonymous mutations avoiding stop codons in the +2 frame (+1 frame once this fragment is inserted in the virus after BP and LR), and attB2 (File S1).

The infectious cDNA clone of CocMoV was built by using a recombination method in yeast, as previously described (56). A detailed description of the methodology is included in the File S2, and the sequence of the CocMoV full-length clone, termed pCocMoV_Su12-25r, was deposited in the NCBI (GenBank accession no. OL744323). For the introduction of an extra TS motif in CocMoV, we modified the pCocMoV_Su12-25r plasmid by taking advantage of its unique *Spe*I restriction site located at the CocMoV P1 coding sequence. Inserts were first generated as small double-stranded DNAs by *in vitro* annealing of specific oligonucleotide pairs (#1/#2 and #3/#4) and then introduced into the *SpeI*-linearized plasmid by ligation to get the corresponding pCocMoV_Su12-25r derivatives.

The PVX-derived constructs pGWC-PVX-eGFP, pGWC-PVX-HF_slip_eGFP, and pGWC-PVX-HF_slip_mut_eGFP were built by LR recombination, using the previously described pGWC-PVX (37) as the destination vector, and pENTR1A-eGFP, pENTR1A-HF_slip_eGFP and pENTR1A-HF_slip_mut_eGFP as entry clones, respectively. The constructs were prepared by PCR amplification of eGFP from pLONG-GFP (58) with primer pairs #5/#8, #6/#8, and #7/#8 to generate ATG_eGFP_stop, ATG_HF_slip_eGFP_stop, and ATG_HF_slip_mut_eGFP_stop fragments, respectively, and further blunt-end ligation of these products into pENTR1A previously digested with *Xmn*I and *EcoR*V.

### Virus inoculation

For manual inoculation, 15 µL of the crude extract from systemically infected plants (1 g in 2 mL of 5 mM sodium phosphate, pH 7.2) was finger-rubbed on young leaves of 1-month-old plants previously dusted with carborundum.

For inoculation of WMV-, CocMoV-, and PPV-derived full-length clones, biolistic with HandGun (59) for the first two clones and Helios Gene Gun System (Bio-Rad) (60) for the last clone was done as previously described.

### Transient expression of proteins

Co-expression of either UPF1 or U1D along with the silencing suppressor P14 from pothos latent virus in upper noninoculated leaves of PPV-infected *N. benthamiana* plants was carried out by infiltration of *A. tumefaciens* strains harboring the corresponding plamids (36). To do that, we prepared cell cultures and the indicated mixes of agrobacteria as previously described (61).

### RNA extraction, next-generation sequencing, and data analyses

Upper noninoculated leaves of plants infected with the indicated viruses were used to extract total RNA with TRI Reagent (Sigma), as indicated by the manufacturer. Then, 1 µg of total RNA was used to generate either amplicons or cDNA libraries for next-generation sequencing.

To generate amplicons, RNA samples (from three independent plants in all cases) were subjected to reverse transcription with the AMV enzyme (Promega) and random hexanucleotides as primers, and the so-generated cDNAs were then used as the template for the PCR amplification of 250-to-300-nt fragments spanning the indicated slippage motifs. Primer pairs #9/#10 and #11/#12 were used to get amplicons spanning the PIPO and the artificially introduced slippage motifs in PPV, respectively. Primer pairs #13/#14, #15/#16 and #17/#16 were used to get amplicons spanning the naturally occurring, as well as a long and a short fragment of artificially introduced slippage motifs in PVX, respectively. Primer pairs #18/#19, #20/#21, and #22/#23 were used to get amplicons spanning PIPO, P1-ALT, and the artificially introduced slippage motifs in CocMoV, respectively. Primer pairs #24/#25 and #26/#27 were used to get amplicons spanning PIPO and PISPO in sweet potato virus 2 (SPV2), respectively. Finally, primers #28/#29, #30/#31, and #32/#33 were used to get amplicons spanning PIPO in watermelon mosaic virus WMV, CVYV, and WMVBV, respectively. These RT-PCR products were sent to the genomics facility at Madrid Science Park. Paired-end sequencing (2 × 300) was done with MiSeq Reagent Kit v3 in a MiSeq platform (Illumina) by following the manufacturer’s instructions.

To analyze the transcriptome of an areca palm leaf infected with ANSSV, total RNA was sent to Biowefind Biotechnology Co., Ltd. (Wuhan, China). After rRNA depletion, RNA was used to prepare the library with the NEBNext Ultra Directional RNA Library Prep Kit, which was further sequenced with a NovaSeq 6000 platform (Illumina) by following the manufacturer’s instructions.

Sequencing reads were aligned against the genome sequence of their corresponding viruses with bowtie2 (62). These alignments were analyzed with SAMtools mpileup (63) to further generate lists of single-nucleotide polymorphisms, as well as small insertions/deletions with their corresponding frequencies at each position, by using in-house Perl scripts (available upon request). Statistical differences between TS rates were tested with the *post-hoc* Tukey HDS test.

### Fluorescence imaging and quantification

GFP fluorescence in infected plants was observed with an epifluorescence stereomicroscope using excitation and barrier filters at 470/40 nm and 525/50 nm, respectively, and photographed with an Olympus DP70 digital camera. The fluorescence intensity was estimated using ImageJ software.

### Detection of proteins by Western blot

Preparation of protein samples, separation on SDS-PAGE, and the electroblotting to nitrocellulose membranes were done as previously described (64). Ponceau red was used to verify equivalent loading of total proteins in each sample. PPV was detected using anti-CP rabbit serum as the primary antibody and horseradish peroxidase (HRP)-conjugated goat anti-rabbit IgG (Jackson ImmunoResearch) as the secondary reagent. GFP was detected using a mixture of two anti-GFP monoclonal antibodies (Roche Applied Science) as primary detection reagents and HRP-conjugated sheep anti-mouse IgG (Amersham) as the secondary reagent. The immunostained proteins were visualized by enhanced chemiluminescence detection with Clarity ECL Western blotting substrate (Bio-Rad) in a ChemiDoc apparatus (Bio-Rad).

## SUPPLEMENTAL MATERIAL

File S1 mbio.02120-24-s0001.docx Sequences.

File S2 mbio.02120-24-s0002.docx Generation of a CocMoV infectious clone.

Fig. S1 mbio.02120-24-s0003.tiff RNA polymerase slippage in CocMoV.

Fig. S2 mbio.02120-24-s0004.tiff Fluorescence quantification in leaf tissues of plants infected with GFP-tagged PPV variants.

Fig. S3 mbio.02120-24-s0005.tiff Conservation of the U8 motif in isolates of WMVBV.

Fig. S4 mbio.02120-24-s0006.tiff Effect of co-infections on CocMoV TS rates.

Fig. S5 mbio.02120-24-s0007.tiff Fluorescence quantification in leaf tissues of plants infected with GFP-tagged PVX variants.

Legends mbio.02120-24-s0008.docx Legends for supplemental figures.

Table S1 mbio.02120-24-s0009.xlsx Viruses from the *Potyviridae* family with fully sequenced genomes.

Table S2 mbio.02120-24-s0010.docx Oligonucleotides/primers used in this study.

ASM does not own the copyrights to Supplemental Material that may be linked to, or accessed through, an article. The authors have granted ASM a non-exclusive, world-wide license to publish the Supplemental Material files. Please contact the corresponding author directly for reuse.
