# Supplementary material for: Reconceptualizing transcriptional slippage in plant RNA viruses
Source: mBio. 2024 Sep 17;15(10):e02120-24. doi: 10.1128/mbio.02120-24 (PMC11481541; doi:10.1128/mbio.02120-24)
Supplement: File S1 — Sequences. [file mbio.02120-24-s0001.docx]

**>attB1-HF_slip-eGFP-attB2**

GGGGACAAGTTTGTACAAAAAAGCAGGCTC***CGCGCGGAAAAAACGCGCGGA***ATGGTGTCAAAGGGCGAGGAGCTGTTCACCGGGGTGGTGCCCATCCTGGTCGAGCTGGACGGCGACGTTAACGGCCACAAGTTCAGCGTGTCCGGCGAGGGCGAGGGCGATGCCACCTACGGCAAGCTCACCCTCAAGTTCATCTGCACCACCGGCAAGCTGCCCGTGCCCTGGCCCACCCTCGTTACCACCTTCACCTACGGCGTGCAGTGCTTCAGCCGCTACCCCGACCACATGCGACAGCACGACTTCTTCAAGTCCGCCATGCCCGAAGGCTACGTCCAGGAGCGCACCATCTTCTTCAAAGACGACGGCAACTACAAGACCCGCGCCGAGGTTAAGTTCGAGGGCGACACCCTGGTTAACCGCATCGAGCTCAAGGGCATCGACTTCAAGGAGGACGGCAACATCCTGGGGCACAAGCTGGAGTACAACTACAACAGCCACAACGTCTATATCATGGCCGACAAGCAGAAGAACGGCATCAAGGTTAACTTCAAGATCCGCCACAACATCGAGGACGGCAGCGTGCAGCTCGCCGACCACTACCAGCAGAACACCCCCATCGGCGACGGCCCCGTGCTGCTGCCCGACAACCACTACCTTAGCACCCAGTCCGCCCTTAGCAAAGACCCCAACGAGAAGCGCGATCACATGGTCCTGCTGGAGTTCGTTACCGCCGCCGGGATCACTCACGGCATGGACGAGCTGTACAAACCCAGCTTTCTTGTACAAAGTGGTCCCC

**>attB1-HF_slip_mut-eGFP-attB2**

GGGGACAAGTTTGTACAAAAAAGCAGGCTC***CGCGCGGAGAAGACGCGCGGA***ATGGTGTCAAAGGGCGAGGAGCTGTTCACCGGGGTGGTGCCCATCCTGGTCGAGCTGGACGGCGACGTTAACGGCCACAAGTTCAGCGTGTCCGGCGAGGGCGAGGGCGATGCCACCTACGGCAAGCTCACCCTCAAGTTCATCTGCACCACCGGCAAGCTGCCCGTGCCCTGGCCCACCCTCGTTACCACCTTCACCTACGGCGTGCAGTGCTTCAGCCGCTACCCCGACCACATGCGACAGCACGACTTCTTCAAGTCCGCCATGCCCGAAGGCTACGTCCAGGAGCGCACCATCTTCTTCAAAGACGACGGCAACTACAAGACCCGCGCCGAGGTTAAGTTCGAGGGCGACACCCTGGTTAACCGCATCGAGCTCAAGGGCATCGACTTCAAGGAGGACGGCAACATCCTGGGGCACAAGCTGGAGTACAACTACAACAGCCACAACGTCTATATCATGGCCGACAAGCAGAAGAACGGCATCAAGGTTAACTTCAAGATCCGCCACAACATCGAGGACGGCAGCGTGCAGCTCGCCGACCACTACCAGCAGAACACCCCCATCGGCGACGGCCCCGTGCTGCTGCCCGACAACCACTACCTTAGCACCCAGTCCGCCCTTAGCAAAGACCCCAACGAGAAGCGCGATCACATGGTCCTGCTGGAGTTCGTTACCGCCGCCGGGATCACTCACGGCATGGACGAGCTGTACAAACCCAGCTTTCTTGTACAAAGTGGTCCCC

**>attB1-CocMoV_*alt*_slip-eGFP-attB2**

GGGGACAAGTTTGTACAAAAAAGCAGGCTC***CCCAAGGAAAAAATTCAAAAG***ATGGTGTCAAAGGGCGAGGAGCTGTTCACCGGGGTGGTGCCCATCCTGGTCGAGCTGGACGGCGACGTTAACGGCCACAAGTTCAGCGTGTCCGGCGAGGGCGAGGGCGATGCCACCTACGGCAAGCTCACCCTCAAGTTCATCTGCACCACCGGCAAGCTGCCCGTGCCCTGGCCCACCCTCGTTACCACCTTCACCTACGGCGTGCAGTGCTTCAGCCGCTACCCCGACCACATGCGACAGCACGACTTCTTCAAGTCCGCCATGCCCGAAGGCTACGTCCAGGAGCGCACCATCTTCTTCAAAGACGACGGCAACTACAAGACCCGCGCCGAGGTTAAGTTCGAGGGCGACACCCTGGTTAACCGCATCGAGCTCAAGGGCATCGACTTCAAGGAGGACGGCAACATCCTGGGGCACAAGCTGGAGTACAACTACAACAGCCACAACGTCTATATCATGGCCGACAAGCAGAAGAACGGCATCAAGGTTAACTTCAAGATCCGCCACAACATCGAGGACGGCAGCGTGCAGCTCGCCGACCACTACCAGCAGAACACCCCCATCGGCGACGGCCCCGTGCTGCTGCCCGACAACCACTACCTTAGCACCCAGTCCGCCCTTAGCAAAGACCCCAACGAGAAGCGCGATCACATGGTCCTGCTGGAGTTCGTTACCGCCGCCGGGATCACTCACGGCATGGACGAGCTGTACAAACCCAGCTTTCTTGTACAAAGTGGTCCCC

**>attB1-CocMoV_*pipo*_slip-eGFP-attB2**

GGGGACAAGTTTGTACAAAAAAGCAGGCTC***CATTGAGAAAAAAACACGATT*** ATGGTGTCAAAGGGCGAGGAGCTGTTCACCGGGGTGGTGCCCATCCTGGTCGAGCTGGACGGCGACGTTAACGGCCACAAGTTCAGCGTGTCCGGCGAGGGCGAGGGCGATGCCACCTACGGCAAGCTCACCCTCAAGTTCATCTGCACCACCGGCAAGCTGCCCGTGCCCTGGCCCACCCTCGTTACCACCTTCACCTACGGCGTGCAGTGCTTCAGCCGCTACCCCGACCACATGCGACAGCACGACTTCTTCAAGTCCGCCATGCCCGAAGGCTACGTCCAGGAGCGCACCATCTTCTTCAAAGACGACGGCAACTACAAGACCCGCGCCGAGGTTAAGTTCGAGGGCGACACCCTGGTTAACCGCATCGAGCTCAAGGGCATCGACTTCAAGGAGGACGGCAACATCCTGGGGCACAAGCTGGAGTACAACTACAACAGCCACAACGTCTATATCATGGCCGACAAGCAGAAGAACGGCATCAAGGTTAACTTCAAGATCCGCCACAACATCGAGGACGGCAGCGTGCAGCTCGCCGACCACTACCAGCAGAACACCCCCATCGGCGACGGCCCCGTGCTGCTGCCCGACAACCACTACCTTAGCACCCAGTCCGCCCTTAGCAAAGACCCCAACGAGAAGCGCGATCACATGGTCCTGCTGGAGTTCGTTACCGCCGCCGGGATCACTCACGGCATGGACGAGCTGTACAAACCCAGCTTTCTTGTACAAAGTGGTCCCC

attB1 and attB2 are underlined.

21-nt slippage motifs are in italic and bold fonts.

The coding sequence of a synthetic eGFP, which has silent mutations to avoid stop codons in the +2 frame, is highlighted in green.
