# Supplementary material for: Reconceptualizing transcriptional slippage in plant RNA viruses
Source: mBio. 2024 Sep 17;15(10):e02120-24. doi: 10.1128/mbio.02120-24 (PMC11481541; doi:10.1128/mbio.02120-24)
Supplement: File S2 — Generation of a CocMoV infectious clone. [file mbio.02120-24-s0002.docx]

**Generation of a CocMoV infectious clone**

Five partially overlapping DNA fragments spanning the whole CocMoV genome (GenBank accession no. KU935732) were obtained by RT-PCR. For these amplifications, five primer pairs (listed below) were used along with total RNA, as template, from melon plants infected with CocMoV. In parallel, the intron 2 of the ST-LS1 gene from *Solanum tuberosum* and intron 2 of the NiR gene from *Phaseolus vulgaris* {Johansen, 2008 #67} were amplified by PCR from a previously built WMV infectious clone {Desbiez, 2012 #46}. The two primer pairs used to amplify these introns (listed below) harbour tails at their 5’ end overlapping with CocMoV P3 and CocMoV CI coding sequences, respectively. Therefore, when the plasmid is assembled, the presence of these introns will prevent the potential leaky expression and toxicity of these viral proteins in bacteria. All the amplified fragments along with a linearized plasmid backbone, which was previously used by Desbiez and collaborators for the construction of other infectious clones, were introduced in yeast for recombination as previously described {Desbiez, 2012 #46}. DNA from diverse yeast colonies was extracted and used to transform *E. coli* by conventional methods. Plasmids were further purified, analysed by digestion with restriction enzymes, and used to inoculate melon plants by biolistic as described {Gal-On, 1997 #68}. Melon plants developing the typical viral symptoms induced by CocMoV were indicative of those plasmids carrying the right cDNA sequence of the full-length viral genome. One of these clones, pCocMoV_Su12-25r, was fully sequenced (GenBank accession no. OL744323) and used for this study.

**List of primers used to build pCocMoV_Su12-25r**

| Primer | Sequence (5’-3’) |
| --- | --- |
| CocMoV-1-F | ATATAAGGAAGTTCATTTCATTTGGAGAGGAAAATAAAAATGACATGAAATTTTAC |
| CocMoV-2727-R | AAGGCGCTTGGTGTGCAGC |
| CocMoV-2727-IntronA | AATCAAAACATGCTGCACACCAAGCGCCTTGTAAGTTTCTGCTTCTACC |
| CocMoV-2728-IntronA-R | CCATACACTTTGGCCTTCCGTGCATAAAGCCACCTGCATATCAACAAATTTTG |
| CocMoV-2728-F | GTGGCTTTATGCACGGAAGG |
| CocMoV-3777-R | CCACTTGTGGTCGGCTCTTGAG |
| CocMoV-3777-IntronB-F | TTTGACGACTCAAGAGCCGACCACAAGTGGGTAAGTATGCACTTAAAGA |
| CocMoV-3778-IntronB-R | CGCTTTTCCCCTATGTAGAGTTAGAAGTGGTCTGCATAATTTCAAAGATTG |
| CocMoV-3779-F | ACCACTTCTAACTCTACATAG |
| CocMoV-5800-R | ATGAAAGACACGCTACTCCTAC |
| CocMoV-5700-F | CCGACTGGAGGGTCGTGGC |
| CocMoV-7800-R | CATCGATCGTTAAGATTGG |
| CocMoV-7700-F | GATTCGGTCATTTCGACTGG |
| CocMoV-R | GCGAATCTAGATTTTTTTTTTTTTTTTTTTTGGAATTTTACGCATAAGGGGTTAAT |

Sequences aligning with the backbone are highlighted in green.

Sequences aligning with introns are highlighted in yellow.
