# Supplementary figures and images for: Reconceptualizing transcriptional slippage in plant RNA viruses
Source: mBio. 2024 Sep 17;15(10):e02120-24. doi: 10.1128/mbio.02120-24 (PMC11481541; doi:10.1128/mbio.02120-24)

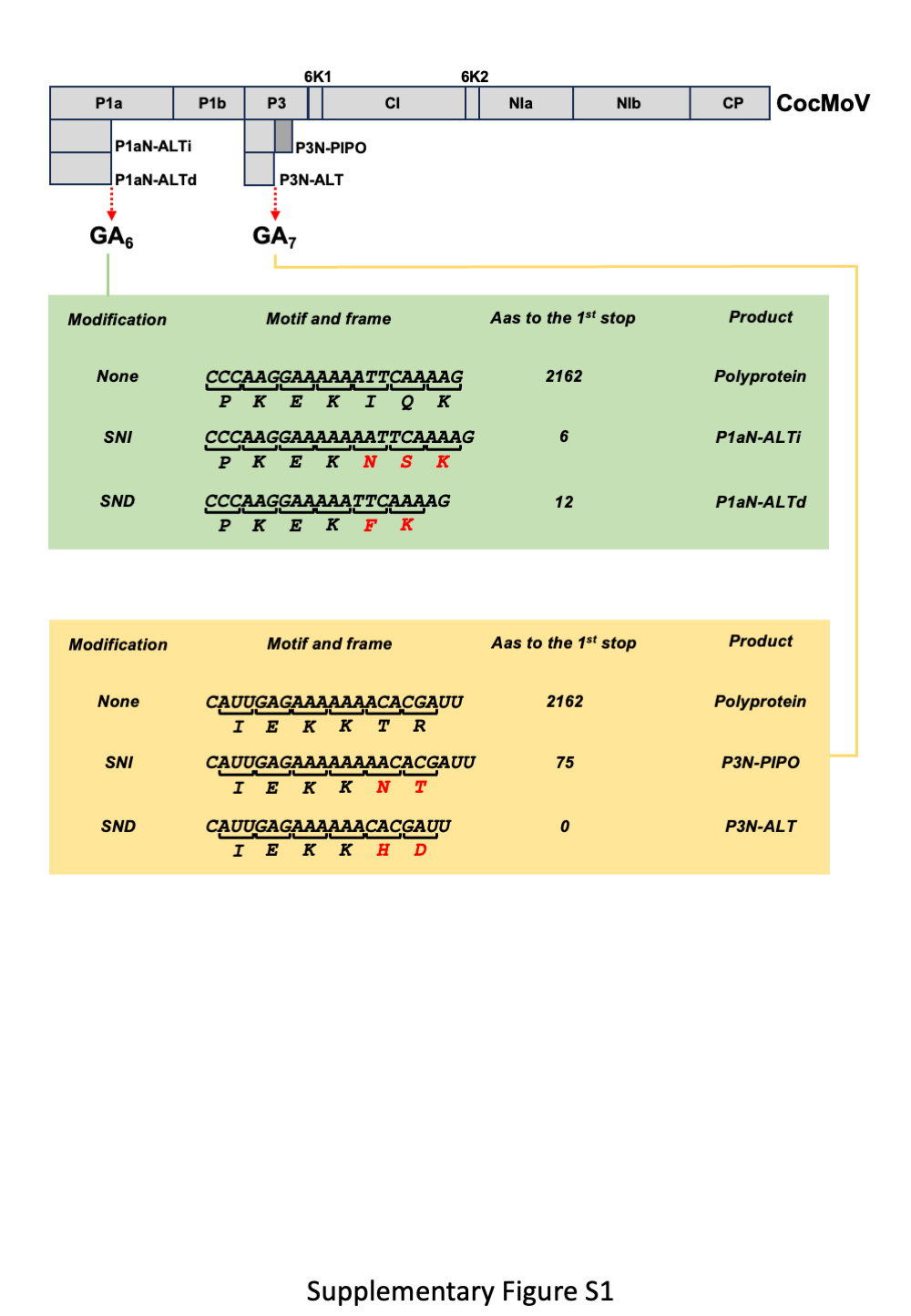

Supplement: Fig. S1 — RNA polymerase slippage in CocMoV. [file mbio.02120-24-s0003.tiff]

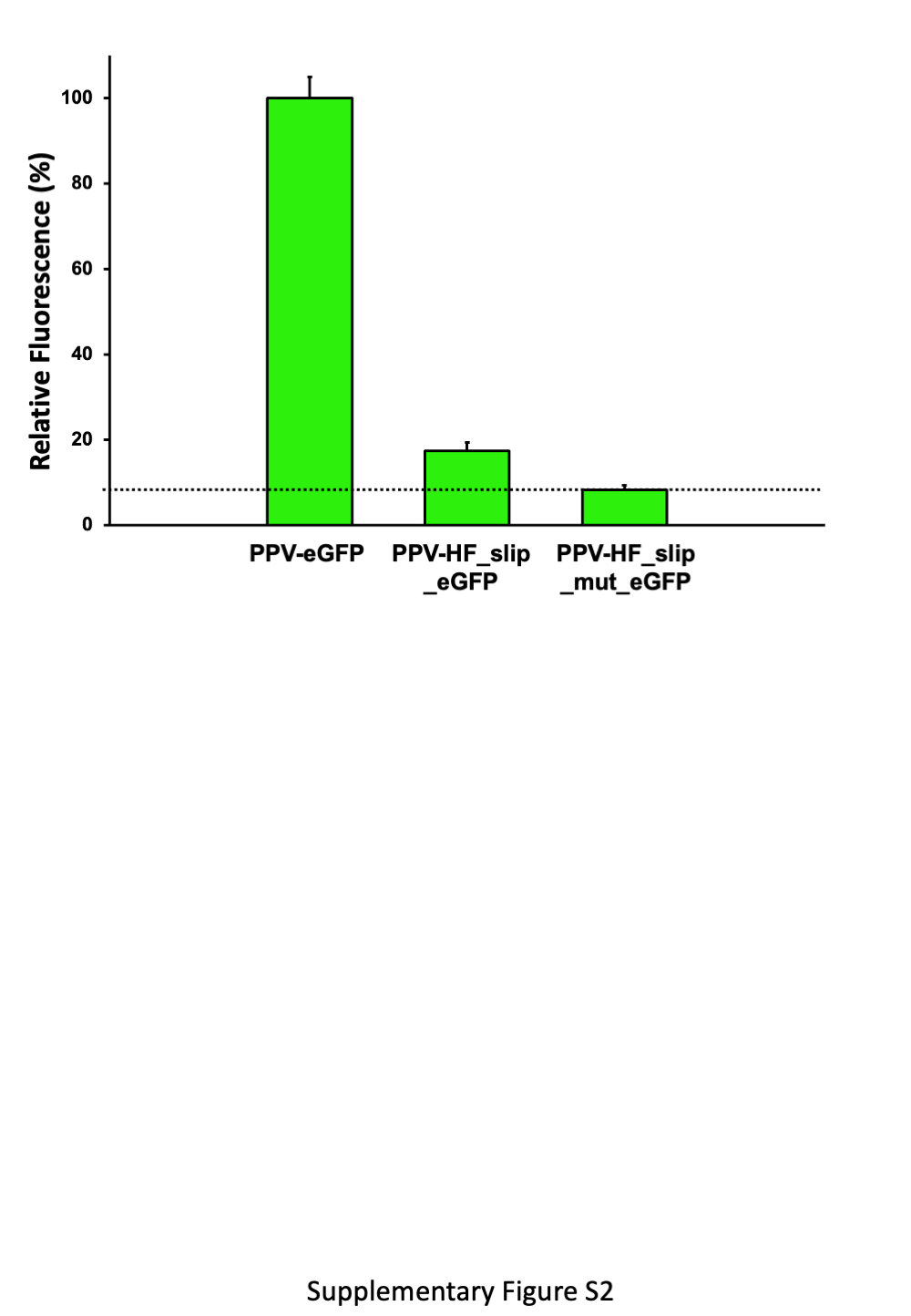

Supplement: Fig. S2 — Fluorescence quantification in leaf tissues of plants infected with GFP-tagged PPV variants. [file mbio.02120-24-s0004.tiff]

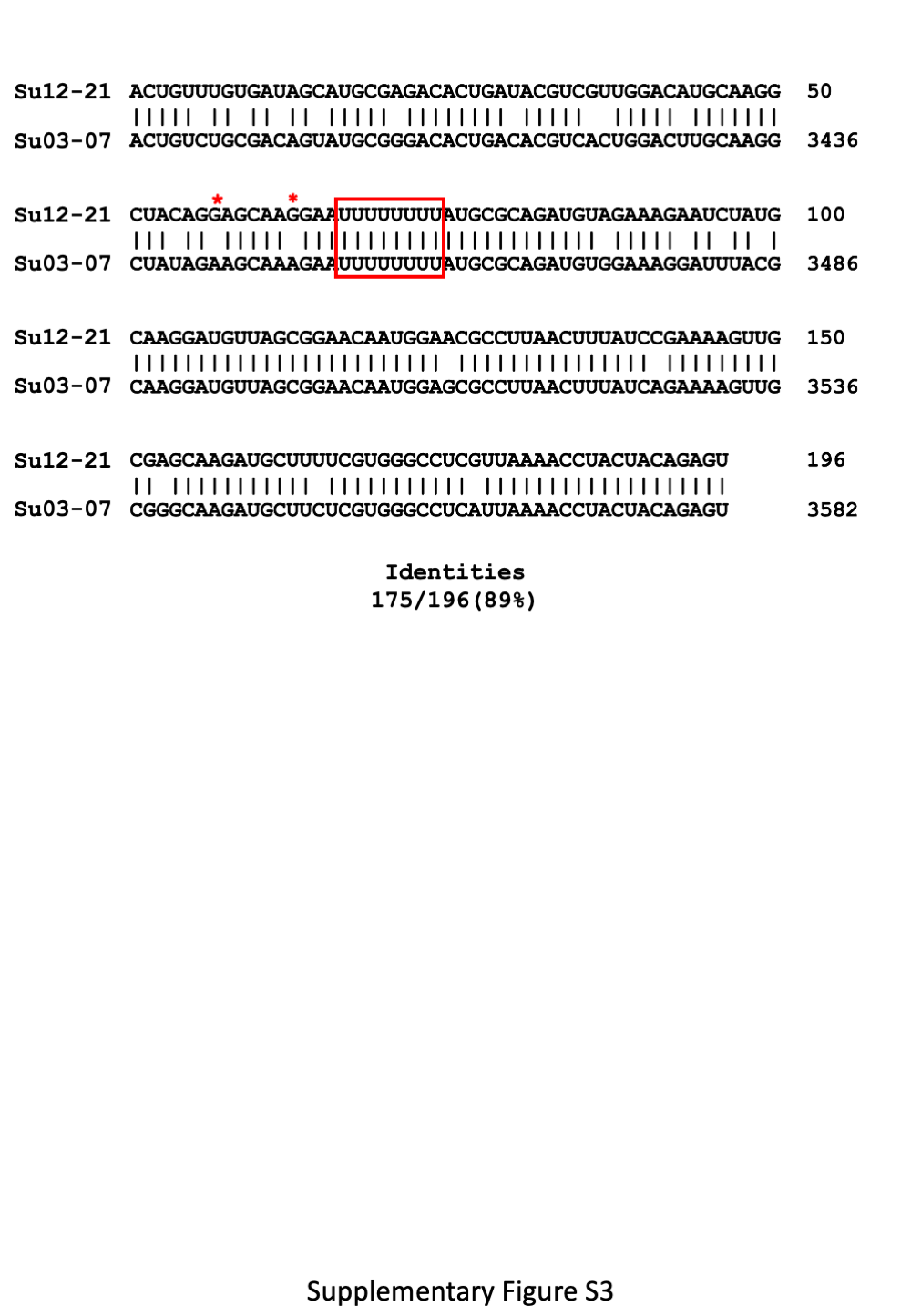

Supplement: Fig. S3 — Conservation of the U8 motif in isolates of WMVBV. [file mbio.02120-24-s0005.tiff]

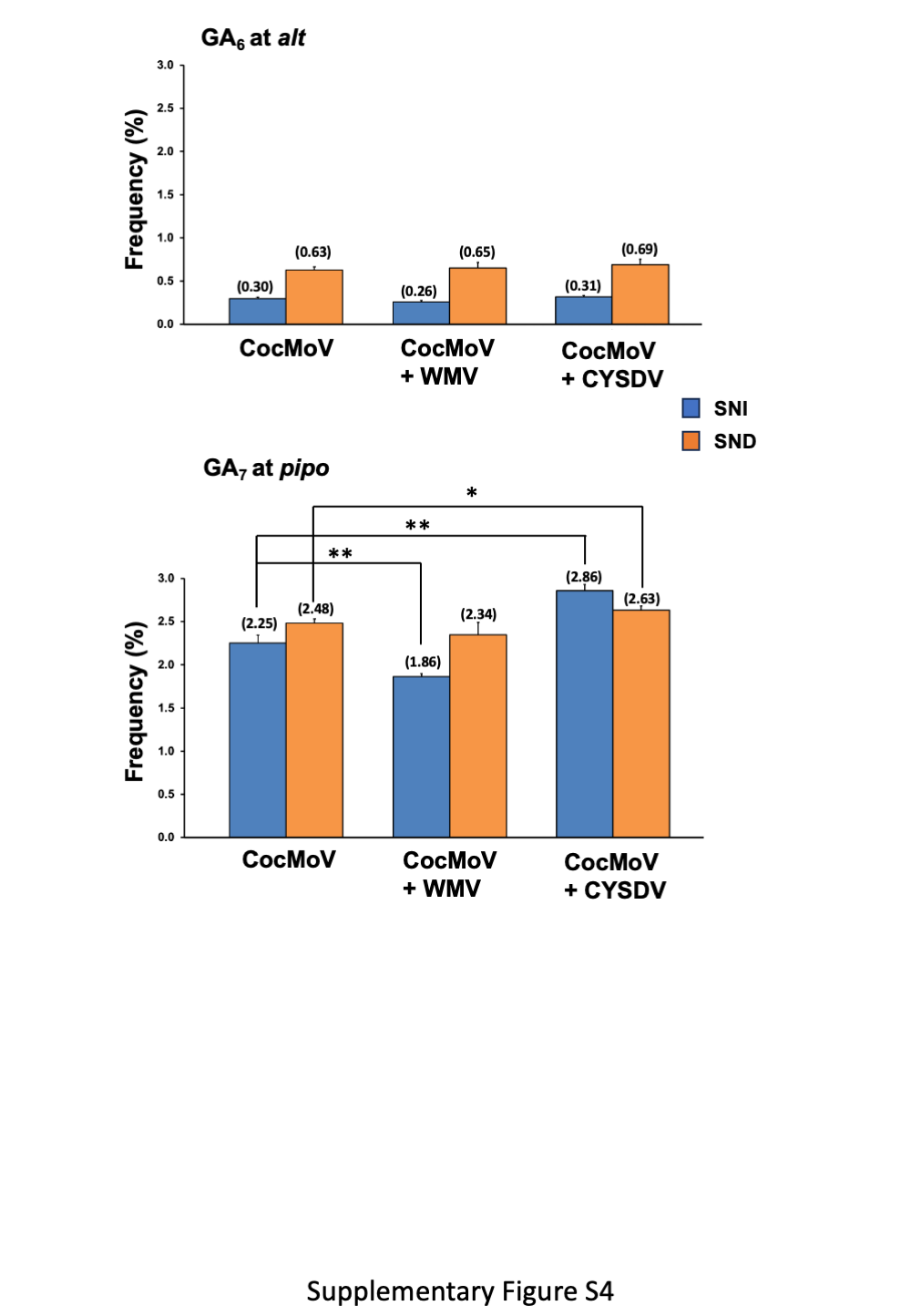

Supplement: Fig. S4 — Effect of co-infections on CocMoV TS rates. [file mbio.02120-24-s0006.tiff]

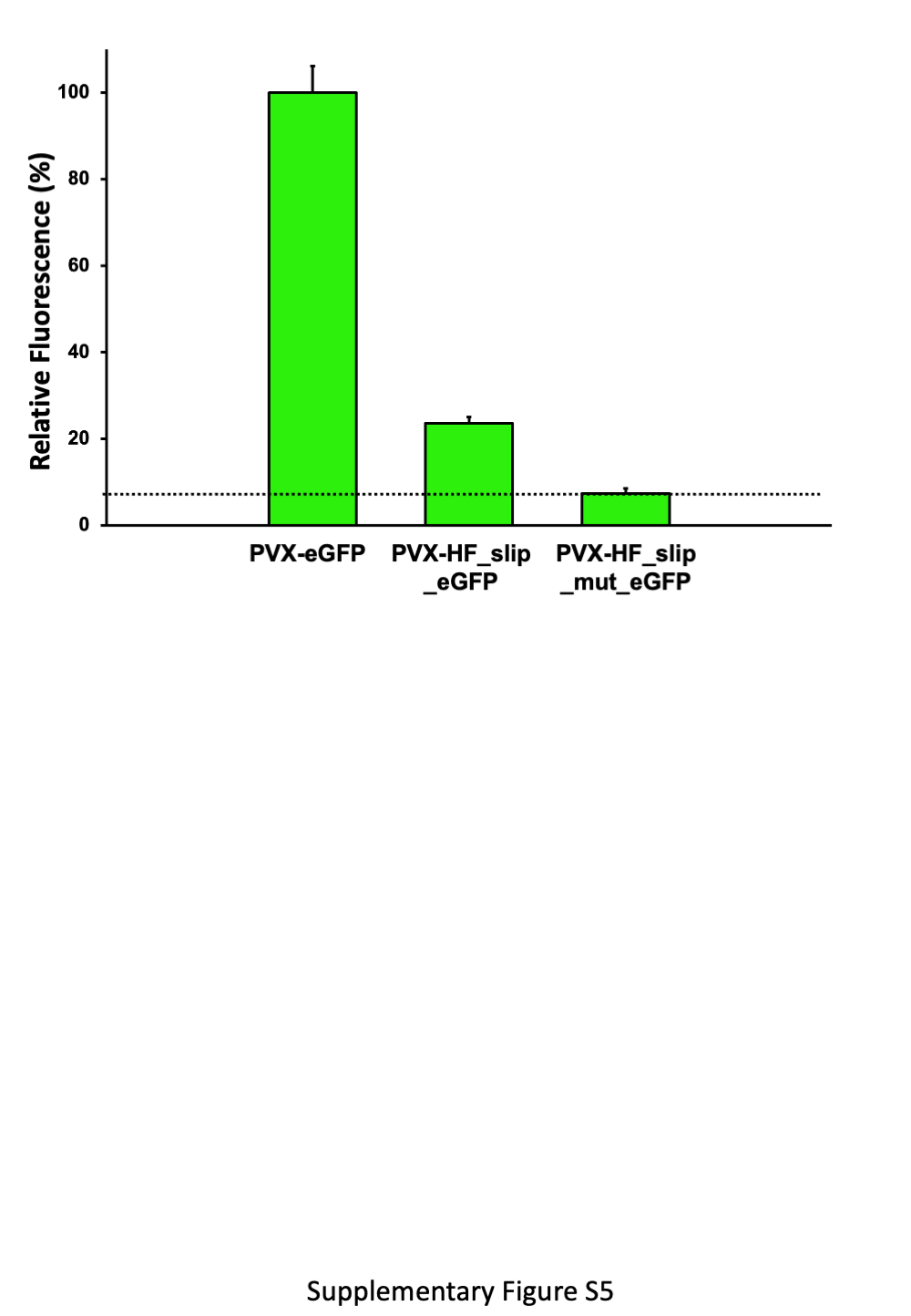

Supplement: Fig. S5 — Fluorescence quantification in leaf tissues of plants infected with GFP-tagged PVX variants. [file mbio.02120-24-s0007.tiff]
