# Supplementary material for: Reconceptualizing transcriptional slippage in plant RNA viruses
Source: mBio. 2024 Sep 17;15(10):e02120-24. doi: 10.1128/mbio.02120-24 (PMC11481541; doi:10.1128/mbio.02120-24)
Supplement: Legends — for supplemental figures. [file mbio.02120-24-s0008.docx]

**Legends for Supplementary Figures**

**Supplementary Figure S1. RNA polymerase slippage in CocMoV.** Schematic representation of the CocMoV coding sequence. The two slippage motifs in the viral genome are indicated, along with the resulting frames in case that no modification (None), single nucleotide insertion (SNI) or single nucleotide deletion (SND) occurs. The names and lengths of the expected products for each case are also indicated.

**Supplementary Figure S2. Fluorescence quantification in leaf tissues of plants infected with GFP-tagged PPV variants.** Bars represent the average fluorescence intensity for equivalent areas of leaf tissues systemically infected with the indicated viruses. Results are expressed relative to PPV-eGFP, which is set to 100% intensity. Error bars represent the standard deviation (n = 4). The dotted line indicates the background autofluorescence of leaf tissue. These tissues correspond to those analysed in Figure 2.

**Supplementary Figure S3. The conservation of the U_8_ motif in isolates of WMVBV.** Alignment of a segment from the P3 coding sequence from Su12-21 and the genome sequence from Su03-07, two WMVBV isolates. The presence of a conserved U_8_ motif is highlighted by a red square. Two specific nucleotide differences between these isolates are indicated by red asterisks.

**Supplementary Figure S4. The effect of co-infections on CocMoV TS rates.** Slippage frequencies at the GA_6_ motif in *alt* (upper panel) and at the GA_7_ motif located upstream of the *pipo* ORF in CocMoV (bottom panel). Bars represent the average frequencies (indicated between brackets) for single nucleotide insertions (SNI) and single nucleotide deletions (SND) at the indicated slippage motifs. Error bars represent standard deviation (n = 3). Statistical differences were tested with the post-hoc Tukey HDS test (* *p* value < 0.05; ** *p* value < 0.01).

**Supplementary Figure S5. Fluorescence quantification in leaf tissues of plants infected with GFP-tagged PVX variants.** Bars represent the average fluorescence intensity for equivalent areas of leaf tissues systemically infected with the indicated viruses. Results are expressed relative to PVX-eGFP, which is set to 100% intensity. Error bars represent the standard deviation (n = 4). The dotted line indicates the background autofluorescence of leaf tissue. These tissues correspond to those analysed in Figure 2.
