# Supplementary material for: Reconceptualizing transcriptional slippage in plant RNA viruses
Source: mBio. 2024 Sep 17;15(10):e02120-24. doi: 10.1128/mbio.02120-24 (PMC11481541; doi:10.1128/mbio.02120-24)
Supplement: Table S2 — Oligonucleotides/primers used in this study. [file mbio.02120-24-s0010.docx]

**Supplementary table S2**. Oligonucleotides/primers used in this study.

| **Primer** | **Sequence (5’-3’)** |
| --- | --- |
| #1 | CTAGCGCGCGGAAAAAACGCGCGG |
| #2 | CTAGCCGCGCGTTTTTTCCGCGCG |
| #3 | CTAGTTGGTGGAAAAAAGTTATCT |
| #4 | CTAGAGATAACTTTTTTCCACCAA |
| #5 | ATGGTGAGCAAGGGCGAGGAGCTG |
| #6 | ATGGCACGCGCGGAAAAAACGCGCGGAAGGTGAGCAAGGGCGAGGAGCT |
| #7 | ATGGCACGCGCGGAGAAGACGCGCGGAAGGTGAGCAAGGGCGAGGAGCT |
| #8 | TTACTTGTACAGCTCGTCCATGCC |
| #9 | ATGATGGCAGTGAAAACTTTG |
| #10 | TTTTGGGATGAAAGGTTTTGG |
| #11 | AAGCTTTACACTGACACTGAAGC |
| #12 | AACTTGTGGCCGTTAACGTC |
| #13 | AACCTGACAGGTACGTGATTCC |
| #14 | AGGGTCTTTGTTAGCAAGCC |
| #15 | TCCGTTGAACGGTTAAGTTTCC |
| #16 | TTGCCGGTGGTGCAGATGAAC |
| #17 | AACAAGTTTGTACAAAAAAGCAGG |
| #18 | AGAAGATGAACCAGAACTGTCAAG |
| #19 | CGTTTTCTTGCTTTTCCCATAAAC |
| #20 | TTGCGTCAGAAAGGATGTTGTGG |
| #21 | GCGGGGTTGATAATGAAGTCACC |
| #22 | GATGAAGAGAAGGTTCGGGACTG |
| #23 | CACTCGCACTCAAACTCATCC |
| #24 | CTTCTTATCTGATCGATAACTTGTGG |
| #25 | CAAACCTCCTATATTTACTTGGGG |
| #26 | TGGGGCGATCGGAAAACTACACC |
| #27 | AGTGGCTTCCTCCATATGACTCG |
| #28 | AGCAAAGGATCTTTTGGCTATG |
| #29 | CACTCACAAAGTTTCTTGAATATG |
| #30 | AATGTCCAAGATGAACCAGAGCTAG |
| #31 | TTCCCATAAATCGTACAAACGCGTC |
| #32 | TCTTGACAGGCAGTCAAAACC |
| #33 | TGGGATTAAATACTCTGTAGTAGG |
